# The Interplay between Host Defense, Infection, and Clinical Status in Septic Patients: A Narrative Review

**DOI:** 10.3390/ijms23020803

**Published:** 2022-01-12

**Authors:** Alessandro Lazzaro, Gabriella De Girolamo, Valeria Filippi, Giuseppe Pietro Innocenti, Letizia Santinelli, Giancarlo Ceccarelli, Enrico Maria Trecarichi, Carlo Torti, Claudio Maria Mastroianni, Gabriella d’Ettorre, Alessandro Russo

**Affiliations:** 1 Department of Public Health and Infectious Diseases, “Sapienza” University of Rome, 00161 Rome, Italy; alessandro.lazzaro@uniroma1.it (A.L.); gabriella.degirolamo@uniroma1.it (G.D.G.); valeria.filippi@uniroma1.it (V.F.); giuseppepietro.innocenti@uniroma1.it (G.P.I.); letizia.santinelli@uniroma1.it (L.S.); giancarlo.ceccarelli@uniroma1.it (G.C.); claudio.mastroianni@uniroma1.it (C.M.M.); gabriella.dettorre@uniroma1.it (G.d.); 2 Infectious and Tropical Disease Unit, Department of Medical and Surgical Sciences, “Magna Graecia” University of Catanzaro, 88100 Catanzaro, Italy; em.trecarichi@unicz.it (E.M.T.); torti@unicz.it (C.T.)

**Keywords:** sepsis, pathogenesis, management, diagnosis, antibiotic therapy

## Abstract

Sepsis is a life-threatening condition that arises when the body’s response to an infection injures its own tissues and organs. Despite significant morbidity and mortality throughout the world, its pathogenesis and mechanisms are not clearly understood. In this narrative review, we aimed to summarize the recent developments in our understanding of the hallmarks of sepsis pathogenesis (immune and adaptive immune response, the complement system, the endothelial disfunction, and autophagy) and highlight novel laboratory diagnostic approaches. Clinical management is also discussed with pivotal consideration for antimicrobic therapy management in particular settings, such as intensive care unit, altered renal function, obesity, and burn patients.

## 1. Introduction

**Figure 1:**
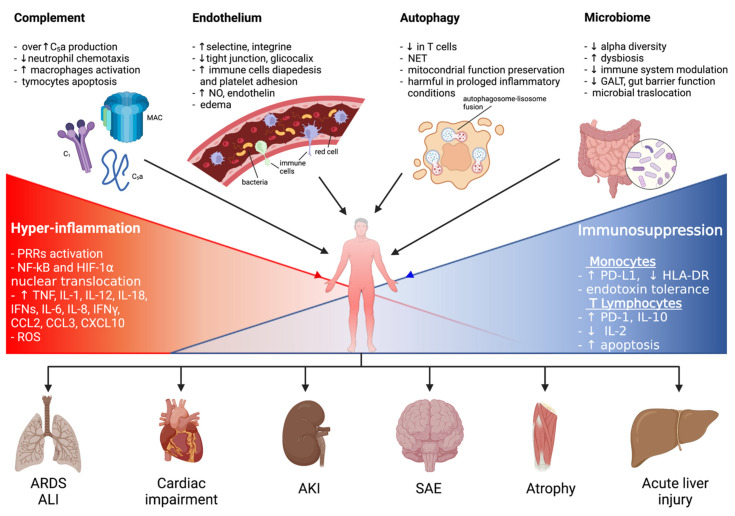
A graphical summary of the pathophysiological mechanisms involved in sepsis onset and persistence and related organ injuries. ALI: acute lung injury; AKI: acute kidney injury; ARDS: acute respiratory distress syndrome; CCL: C-C motif chemokine ligand; CXCL: C-X-C motif chemokine ligand; ET: endotoxin tolerance; GALT: gut-associated lymphoid tissue; HIF-1α: hypoxia-inducible factor-1α; HLA-DR: human leukocyte antigen-DR isotype; IFNγ: interferon γ; IL: interleukin; LPS: lipopolysaccharide; MAC: membrane attack complex; NET: neutrophil extracellular trap; NF-kB: nuclear factor kappa-light-chain-enhancer of activated B cells; NO: nitric oxide; PD-1: programmed cell death 1 receptor; PD-L1: programmed cell death ligand 1; PRRs: pattern recognition receptors; ROS: reactive oxygen species; SAE: sepsis-associated encephalopathy.

Sepsis is an important syndrome associated with significant morbidity and mortality. The true extent of sepsis is not fully understood due to its variability and the lack of specific epidemiological data, due to different diagnostic criteria and definitions. The World Health Organization (WHO) has stated that the worldwide annual mortality due to sepsis is around 6 million, with most of these deaths being preventable [1,2,3,4,5,6,7]. Originally defined as “the decomposition of animal or vegetable matter in the presence of bacteria” [8], “sepsis” is currently identified as “a life-threatening condition that arises when the body’s response to an infection injures its own tissues and organs” [9]. Initiated by an invading pathogen, generally represented by bacteria and, less frequently, by viruses or fungi, sepsis results in an inflammatory process in which the body’s own response has a deleterious effect upon itself. This pathophysiological response can culminate in multiorgan failure, usually due to a combination of cardiovascular, cellular, coagulation and endothelial dysfunction [10], eventually leading to septic shock, a clinical proinflammatory response, predominantly cytokine-mediated (see Figure 1).

To understand the importance, complexity, and challenges posed by sepsis, this narrative review provides background on the postulated pathophysiological mechanisms underlying sepsis, before focusing on diagnostic and therapeutical considerations about this syndrome.

## 2. Literature Research

The literature research was conducted between March and April 2021 using the well-established PubMed database. Animal models, case series, controlled and uncontrolled studies, and meta-analyses were included. Case reports were excluded. Only English-language papers were taken into account. We did not set a specific time window for the research, but the focus was placed on papers published in the last 10 years. The keywords used were “sepsis”, “septic shock”, “pathogenesis”, “innate immunity”, “adaptive immunity”, “complement system”, “endothelium”, “autophagy”, “diagnosis”, “biomarker”, “antibiotic therapy”, “intensive care unit”, “obesity”, “burn patients”, “pharmacokinetic”, and/or “adjunctive therapies”. In the first step, papers were screened by abstract and title. Then, the full text of the selected papers was examined. Papers were excluded if their content had nothing to do with sepsis pathophysiology, diagnosis, or antimicrobic therapeutical approach.

## 3. Pathophysiology of Sepsis

### 3.1. The Innate Immunity

The mechanisms resulting in the development of sepsis are very complex and not completely understood. However, it is well established that at the beginning of sepsis the inflammatory response is mediated by the activation of the innate immune system cells, mainly represented by macrophages, monocytes, neutrophils, and natural killer cells. Multiple infection-derived microbial products are simultaneously recognized by complement and specific cell-surface receptors. Amongst these, toll-like receptors (TLRs) are transmembrane receptors expressed by monocytes and macrophages and able to detect extracellular pathogen-associated molecular patterns (PAMPs) (such as bacterial endotoxins and fungal β-glucans) and damage-associated molecular patterns (DAMPs) released from injured endogenous cells (such as ATP, high mobility group proteins, and mitochondrial DNA). To this extent, nod-like receptors (NODs), which are expressed intracellularly, recognize pathogens invading the cytosol. Furthermore, retinoic acid inducible gene (RIG)-like receptors, mannose-binding lectin (MBL), and scavenger receptors also take part in this process [11].

Therefore, the binding between cellular receptors and different components of bacteria, viruses, and fungi, as well as host products derived from tissue damage, induces multiple intracellular signaling pathways, ultimately leading to the expression of several common gene classes involved in inflammation, adaptive immunity, and cellular metabolism, the second key step in the activation of the immune response during sepsis. In particular, the phosphorylation of mitogen-activated protein kinases (MAPKs), Janus kinases (JAKs), or signal transducers and activators of transcription (STATs) and nuclear translocation of nuclear factor κΒ (NF-κΒ) initiate the expression of multiple early activation genes which are promptly translated into signaling proteins, including cytokines associated with inflammation (such as tumor necrosis factor (TNF), interleukin (IL)-1, IL-12, IL-18, and type I interferons (IFNs)). These proinflammatory intermediates subsequently induce a cascade of other inflammatory cytokines and chemokines (including IL-6, IL-8, IFNγ, C-C motif chemokine ligand 2 (CCL2), CCL3, and C-X-C motif chemokine ligand 10 (CXCL10)), as well as the polarization and suppression of components of adaptive immunity [12].

These inflammatory networks cause the activation of the complement system, the proliferation of leukocytes, and an increased expression of endothelial adhesion molecules, with profound effects on coagulation and vascular and lymphatic endothelium, such as the transition of the endothelium to a procoagulant state, the loss of endothelial tight junctions, and increased vascular permeability [13].

### 3.2. The Complement System

The complement system, which consists of multiple proteins in body fluids, receptors, and regulatory proteins, carries out a defensive action against infectious agents and acts as an immune sensor, effector, and regulator. Complement activation can be initiated via three different pathways: the classical (including antibodies, C1q, C2, and C4), the alternative (including complement factor B and spontaneous C3 hydrolysis to form C3b), and the lectin pathway (including MBL and ficolins) [14,15]. The common result of these pathways is the cleavage of C3 and C5 to generate anaphylatoxin peptides (i.e., C3a and C5a), C3b, and C5b. C3b is an important phagocytosis-promoting product, whereas C5b interacts with C6–C9 to form the membrane attack complex on cell membranes. C5a, under conditions of regulated production, supplies defensive functions by enhancing chemotactic responses of neutrophils, phagocytosis, and oxidative burst that is involved in killing bacteria [16,17,18].

The role of complement in sepsis pathogenesis might appear ambiguous. On one hand, C3 deficiency, which results in the inhibition of most complement effector functions, clearly increases sepsis-associated mortality in animals [19]; these observations underline the pivotal role of complement as a defense mechanism against invading microbes. Contrariwise, other data have indicated that inhibition of C5a signaling improves the survival of experimental animal models [20,21]. Increased production of C5a, as occurs during sepsis, can lead to adverse systemic consequences. Neutrophils become functionally paralyzed [22], unable to respond chemotactically to C5a, but also to the chemotactic peptide N-formyl-Met-Leu-Phe (fMLP), which is produced by bacteria [23]. Perturbations in signaling pathways of neutrophils cause their incapacity to phosphorylate extracellular signal-regulated kinase 1/2 (ERK1/2) which is a crucial factor in the MAPK signaling cascade involved in neutrophils activation. Under these conditions, neutrophils cannot phosphorylate neutrophil cytosol factor 1 (NCF1), preventing the assembly of nicotinamide adenine dinucleotide phosphate (NADPH) oxidase [22]. In the presence of relatively high levels of C5a, macrophages have potentiated responses [24], which lead to a considerably increased production of proinflammatory mediators such as TNF and various chemokines. Moreover, generation of C5a during sepsis is associated with apoptosis of thymocytes, which has been associated with increased binding of C5a to thymocytes, which is due to upregulated expression of C5a receptor mRNA and protein and to the consequent activation of caspases 3, 4, and 6 [25]. Blockade of C5a in experimental models of sepsis has been shown to be beneficial in various models from different studies. For example, inhibition of C5a by antibodies in a primate model of sepsis markedly attenuated *Escherichia coli*-induced septic shock and the development of adult respiratory distress syndrome [26]. Similarly, the blockade of C5a with antibodies in a murine pneumococcal pneumonia model was highly effective in diminishing the severity of sepsis, favoring cellular and organ protection and improving outcome [27].

### 3.3. The Role of the Endothelium

Sepsis is not only a state of systemic inflammation, but also a state of deregulated hemostasis. Hemostasis is a complex process regulated by the endothelium, soluble plasma molecules, platelets, and leukocytes; it not only is involved in the balance between pro- and anticoagulant forces, but also directs platelet and fibrin clotting to areas of focal vascular injury [28]. Sustained inflammation during severe sepsis drives hemostasis in a condition of deregulation characterized by a prothrombotic and antifibrinolytic state, organ ischemia, and multiple organ dysfunction syndrome.

Sepsis is associated with severe endothelium dysfunction leading to deregulation of vascular reactivity and hemostasis. This damage of the endothelial cells (ECs) is considered pivotal to the progression to organ failure during sepsis. Under normal conditions, the endothelium serves as an anticoagulant surface that regulates the flow of gases, water, solutes, lipids, proteins, and other macromolecules within the microcirculation [29,30]. The endothelium integrity is maintained by the cell cytoskeleton (actin), intercellular adhesion molecules (tight junctions), and numerous supportive proteins. During sepsis, these structures are broken up essentially in response to neutrophil and platelet adhesion, the release of inflammatory mediators, and toxic intermediates. Combined with the increased expression of adhesion molecules (selectins and integrins), the binding of leukocytes to the endothelial surface results in vascular fluid leakage and extravasating leukocyte migration across the endothelial barrier. Although in trauma or localized infection these responses enable platelets and immune cells to reach tissue sites, sepsis causes prolonged and generalized responses that can lead to substantial tissue injury [31].

The glycocalyx is a glycoprotein–polysaccharide layer that covers the endothelium and supports several key physiological processes such as vascular barrier function, hemostasis, leukocyte and platelet adhesion, and anti-inflammatory and antioxidant defenses [32,33]. Loss of barrier function induced by glycocalyx shedding, which occurs in the presence of oxidants, cytokines, and bacterial endotoxins [32], is associated with the formation of edema [34] and is a key contributor to sepsis-induced organ failure. Shedding of the glycocalyx may also hamper the ability to sense and transduce blood flood-induced sheer stress, resulting in the endothelial release of nitric oxide (NO) or endothelin (ET). Increased plasma concentrations of NO and ET metabolites are regarded as key mediators in the systemic inflammatory response that lead to fatal multiple organ dysfunction [35].

In sepsis and septic shock, the normal anticoagulative state within the vasculature is disrupted. This state of hypercoagulability is characterized by fibrin deposition, microvascular thrombi, neutrophil extracellular trap formation, and endothelial damage. Platelet activation can itself propagate both coagulation and inflammatory response by forming aggregates that can activate thrombin release, a serine protease that induces secretion of proinflammatory cytokines and growth factors. Platelets might also trigger inflammation by activating dendritic cells [36].

### 3.4. Autophagy

Autophagy is a highly conserved degradative pathway involved in maintaining intracellular homeostasis under physiological conditions, playing a crucial role in the pathogenesis of inflammation and infectious diseases [37,38]. There are three types of autophagy: macroautophagy, chaperone-mediated autophagy, and microautophagy. To eliminate damaged proteins and organelles, as well as cytoplasmatic bacteria and pathogens [39], cells exploit this adaptive mechanism to protect themselves from damages and apoptosis [40]. Several intracellular signaling pathways are responsible for autophagy induction, such as 5′ adenosine monophosphate-activated protein kinase (AMPK) and c-Jun N-terminal kinase (JNK)/p38, two MAPK pathways, generating ROS and regulating the NF-κB under activation of TLR4 and TLR9, respectively [41,42]. Recently, the induction of autophagy has received increased attention in the context of sepsis: it mainly protects the host against multiorgan dysfunction syndrome (MODS) by preventing immune cell apoptosis, maintaining the homeostatic balance between pro- and anti-inflammatory cytokines, and preserving mitochondrial functions [43,44,45,46].

Although the activation of autophagy and high cell vitality should protect cells against microbial infection during the early stages of sepsis, this benefit is limited until severe sepsis occurs, since a substantial increase in autophagy does not reverse the massive inflammatory response established but worsens tissue and organ injury [47,48]. Consistently with these findings, the regulation of autophagy and its mechanism of action during sepsis takes place in different ways. Since sepsis-induced immunosuppression mainly involves the apoptosis of immune cells (such as T cells, macrophages, B cells, and dendritic cells), autophagy interacts with several cellular components to alleviate the excessive inflammatory responses [49]. In the context of acquired immunity, autophagy pathways are involved in the regulation of CD4^+^ T cell apoptosis [50,51]. However, during sepsis this mechanism is insufficient, so the blockade of T cell autophagy accelerates apoptosis; consequently, the increased expression of IL-10 by CD4^+^ T cells might promote a further immunosuppressive state. Lipopolysaccharide (LPS) involvement can increase the macrophage migration inhibitory factor (MIF) secretion rate of autophagy-deficient macrophages, thus worsening inflammation status [52]. A further exacerbation of the inflammatory response occurs when excessive autophagy leads to programmed cell death of macrophages [53]. By contrast, an increase in autophagy induction triggers the formation of neutrophil extracellular traps (NETs), which establish a physical barrier to contain microbes, reducing host tissue damages [54].

A crucial step during sepsis-caused organ failure is represented by mitochondrial dysfunction, which may impair cellular energy and increase oxidative stress [55]. Mitochondria are prone to damage in sepsis, and their inner membrane electrochemical potential decreases: these damaged organelles are removed by autophagosomes and eventually degraded by fusion with lysosomes, promoting the recovery of septic organ function [56]. By contrast, an impairment in the autophagy of mitochondria leads to reduced mitochondrial clearance and an increase in inflammation [57]. Therefore, blocking autophagy promotes mitochondrial damage, increases the production of ROS, elevates inflammatory cytokines, and promotes apoptosis during sepsis. In addition, when sepsis occurs, several noncoding RNAs, such as microRNAs (miRs), can directly regulate autophagy-related proteins and indirectly interfere with autophagy signaling pathways [58,59]. It has been recently demonstrated that the miR-19b-3p protects cells from sepsis-induced inflammation injury via inhibiting the NF-κB signaling pathway, and Krüppel-like factor 7 (KLF7) was a potential target [60]. By contrast, overexpression of miR-126 could protect podocytes from sepsis-induced injury through the epidermal growth factor-like domain multiple 6/dyskeratosis congenita 1 (EGFL6/DKC1) signaling pathway [61].

Based on previous evidence, the protective effects of autophagy are mainly evident on multiple organs and systems, including heart, liver, lungs, kidneys, brain, and coagulation system. In this context, the development of autophagic vacuoles and the expression of autophagy-associated proteins differ between distinct tissues or organs and specific stages of sepsis.

### 3.5. Downregulation of the Immune System

Besides the systemic inflammatory response characterizing sepsis during the early stages of the process, a prolonged state of immunosuppression also occurs in both the initial and late phases of the disease [62,63]. Indeed, patients who survive the early inflammatory stage of sepsis enter a late phase characterized by profound immunosuppression: these patients frequently experience ongoing infectious foci, despite antimicrobial therapy; reactivation of latent viral infection; and acquisition of secondary hospital-acquired infections, often with opportunistic microorganisms, which usually do not tend to infect patients with normal immune status.

Sepsis has been described as a two-phase process where an initial hyperinflammatory phase is followed by a prolonged immunosuppressive phase [64,65]. However, in clinical practice, it is evident that these two phases tend to overlap. Indeed, several studies have shown that both proinflammatory and anti-inflammatory responses occur simultaneously in the first stage of sepsis [66,67,68]. The net effect of such mechanisms results in immunosuppression involving both the innate and the adaptive immune systems.

As is well known from clinical practice, neutrophil count increases in circulating blood within the first hours after sepsis occurs, because of increased release of mature and immature cells from the bone marrow [69] and delayed apoptosis [70]. However, functional abnormalities have been described, including loss of chemotactic activity [71] and production of anti-inflammatory IL-10 able to inhibit lymphocyte proliferation [72,73].

A hallmark of sepsis-induced immunosuppression is represented by the mechanism named “endotoxin tolerance” [74], which concerns monocytes/macrophages, cells involved in both the early and the late phases of sepsis. After the release of the cytokine storm which characterizes the early phase, these cells undergo a reprogramming process involving epigenetic modifications [75] and microRNA activity [76]. After endotoxin tolerance occurs, monocytes/macrophages show reduced ability to release proinflammatory cytokines (TNF, IL-1α, IL-1β, IL-6, IL-12, IFNγ) in response to TLR stimuli, while the production of anti-inflammatory cytokines (IL-1 receptor antagonist and IL-10) seems not impaired or even enhanced [77,78]. The DR isotype of the human leukocyte antigen (HLA-DR) is largely reduced on the cellular surface of monocytes/macrophages, thus accounting for a reduced ability to present antigen to the adaptive immune system [78,79,80,81,82] (this mechanism has also been described among dendritic cells, both myeloid and plasmacytoid). Persisting low monocyte HLA-DR expression predicts mortality in septic shock [83]. A crucial event for the onset of the endotoxin tolerance is represented by the nuclear translocation of the transcription factor hypoxia-inducible factor-1α (HIF-1α), which governs the described reprogramming events through the activation of several genes such as IRAKM, VEGFA, and MMP. Such a process should not be considered as an immune-paralysis outcome, but as a reprogramming process towards cellular activities other than inflammation, such as high phagocytosis activity, tissue remodeling, and antimicrobial activity [74]. Moreover, another effect of HIF-1α activation is the increased expression of the programmed cell death ligand 1 (PD-L1) which leads to lymphocyte apoptosis as described below.

Together with endotoxin tolerance, massive cellular apoptosis of CD4^+^ and CD8^+^ T cells [84] and immune exhaustion of T lymphocytes [65] are typical of sepsis-induced immunosuppression. In the contest of persistent high antigen load, immune exhaustion occurs, a phenomenon characterized by progressive loss of function, changes in transcriptional profiles, and sustained expression of inhibitory receptors. At first, cells lose their ability to produce IL-2 and TNFα, followed by the loss of high proliferative capacity and cytotoxic activity, eventually leading to apoptosis [65]. The programmed cell death 1 (PD1) receptor is highly expressed on the cellular surface of T lymphocytes during immune exhaustion. PD1 activation after the exposure to its ligand PD-L1 (overexpressed by monocytes/macrophages, dendritic cells, and capillary endothelial and bronchial epithelial cells during sepsis) induces intracellular pathways interrupting the T cell receptor transduction signals and ultimately leading to reduction in IL-2 synthesis, inhibition of activation and proliferation, decreased effector functions (cytokine secretion and cytotoxicity), and accelerated apoptosis [85]. PD1 on circulating T cells from patients with sepsis correlated with decreased T cell proliferative capacity, increased nosocomial infections, and mortality [86].

Interestingly, several pieces of evidence pointed out that during sepsis there is a marked reduction in transcription factors which modulate the differentiation towards effector T cells (Tbet, GATA3, and RORγt, respectively modulating the Th1, Th2, and Th17 response), while FOXP3, the transcription factor responsible for differentiation into regulatory T cells (Tregs), is not affected. Indeed, Tregs are not reduced during sepsis and their relative count is expanded, with deleterious effects on T cell proliferation and functions [65,87,88].

### 3.6. The Role of the Microbiome

The microbiome is the microbic consortium of bacteria, viruses, fungi, and protozoa living upon (skin) and inside (gut, lung) our body. The last few decades have seen an increasing interest regarding the physiological and pathogenetic role of the microbiome in several medical conditions, included sepsis. Indeed, the gut microbiome exerts numerous physiological roles as an independent organ within the human body: it produces functionally active metabolites which influence immune functions of several immune cells; it increases the gut barrier function, inhibiting hematic translocations of resident microorganisms; and it directly promotes the maturation of local immune cells, driving the expansion of antigen-specific activated T cells and enhancing responsiveness of immune cells to cytokines [89].

A loss in the alpha diversity, characterized by the reduction in species composing the microbic consortium, has emerged as a negative prognostic factor during sepsis. The imbalance between microbic communities disrupts the inner homeostasis and is capable to lead to an expansion of species with enhanced ability to disseminate to the blood and cause infection [90]; for example, a reduction in anaerobic bacteria in the gut was correlated with overgrowth of aerobic bacteria (such as Staphylococcus and Enterococcus) and an expansion of opportunistic fungi (such as Candida and Aspergillus) [91]. This is particularly true in the case of sepsis, where the prompt use of broad-spectrum antimicrobics is warranted as a first-line regimen, with a consequent reduction in alpha diversity. Moreover, considering that the apoptosis of lymphoid tissues broadly affects the gut-associated lymphoid tissue (both lymph nodes and mucosa-associated lymphoid tissue), it appears evident that sepsis represents an ideal setting for bacterial and fungal translocation, as well for viral reactivation.

Such phenomena are not restricted to the gut, but are of interest in the lung as well, since a progressive reduction in alpha diversity has been described under invasive ventilation until the development of pneumonia [92]. During viral infections, the commensal gut microbiome contributes to set a homeostatic type I IFN-dependent immune response at distal nongastrointestinal tract sites [93,94] and locally regulates NF-kB and inflammasome-dependent release of proinflammatory cytokines (e.g., TNF-α, IL-1β, IL-6, and lL-18) which are pivotal for the distal recruitment of immunocompetent cells (e.g., monocytes, granulocytes, dendritic cells) that circumscribe viral replication [95].

SARS-CoV-2, the novel coronavirus responsible for the COVID-19 pandemic, is not exempt from gut microbiome involvement: indeed, abdominal discomfort, nausea, diarrhea, and vomiting have been described as less common symptoms of COVID-19, with a prevalence of diarrhea of about 10% in some case series. Gut microbiome dysbiosis was observed in hospitalized COVID-19 patients, showing an imbalance of intestinal microflora diversity with decreased levels of probiotic bacteria (e.g., Lactobacillus and Bifidobacterium), a higher relative abundance of opportunistic pathogens (e.g., Streptococcus, Rothia, Actinomyces), and a lower relative abundance of beneficial symbionts. Notably, these shifts in gut microbiome composition persisted after the resolution of respiratory symptoms and were correlated with disease severity [96,97,98].

So far, several efforts have been made to characterize a healthy microbiome, as well as to identify specific microbiome signatures able to predict sepsis onset or higher risk to develop sepsis or to stratify patients with different prognoses [89]. Although there is still a long way to go, currently we know that microbiome composition is associated with susceptibility to sepsis (e.g., intestinal domination with proteobacteria was associated with increased risk of subsequent Gram-negative bloodstream infection among allogeneic hematopoietic cell transplant receivers [99]) and that different enterotypes can predict different outcomes (e.g., intestinal domination of Enterococcus at intensive care unit admission was associated with increased risk for death in patients with and without sepsis [100]).

### 3.7. Cellular, Tissue, and Organ Failure

Sepsis is also described as a systemic disorder, affecting all organs of the body. Although the molecular basis of organ failure remains unclear, six types of organ dysfunction predominantly characterize sepsis: neurological (altered mental status), pulmonary (hypoxemia), cardiovascular (shock), renal (oliguria and/or increased creatinine concentration), hematological (decreased platelet count), and hepatic (hyperbilirubinemia). The underlying mechanism behind tissue and organ dysfunction in sepsis seems to be a diminished oxygen delivery to and utilization by cells with a consequent increased anaerobic glycolysis and lactic acid production. Several factors, including hypotension, reduced red-cell deformability, and microvascular thrombosis, contribute to impair tissue oxygenation in septic shock in addition to mitochondrial damage caused by oxidative stress [101]. All these mechanisms in conjunction with systemic hyperinflammation and sustained immunosuppression, generalized increased catabolism, insulin resistance, and hyperglycemia can contribute to the cellular level damage.

## 4. Diagnosis of Sepsis

### 4.1. MDR and Sepsis

In the last few decades, a specific sepsis population with a high mortality risk is accounted for by patients with septic shock by multidrug-resistant (MDR) microorganisms, with Gram-negative pathogens being responsible for most cases [102]. In particular, an increased frequency of MDR Gram-negative pathogens, such as MDR *Acinetobacter baumannii* (MDR-AB) and Klebsiella pneumoniae carbapenemase-producing *Klebsiella pneumoniae* (KPC-Kp), have been observed among critically ill intensive care unit (ICU) patients.

MDR bacteria are defined by international guidelines as microorganisms nonsusceptible to at least three different antimicrobial categories [103], and the burden of their infection is variable in different areas worldwide [104]. The constantly increasing spread of MDR severe infections is due to a strong increase in the level of care and in the use of antibiotics, despite the improvement in social and health conditions. Although several risk factors for MDR microorganism infections have been identified (see Table 1), the real causes for this increased risk are still unclear, and the appropriateness of initial antibiotic therapy still represents a crucial variable in septic patients, thus affecting the clinical outcome [105,106,107,108].

It is well established that bacterial, viral, fungal, or parasitic infections can cause sepsis, making diagnosis particularly challenging where an infectious source is not immediately obvious. As widely reported in the literature, early recognition of sepsis is critical for timely initiation of treatment. To this extent, patients with severe forms of sepsis treated with antibiotics within 1 h of the onset of symptoms have greater survival chances than those with delayed treatment [109,110]. In this scenario, an early diagnosis of sepsis is crucial to identifying the etiology of infection and a targeted antimicrobial therapy.

### 4.2. Diagnostic Tools

The ideal diagnostic technology for sepsis should include the following characteristics: a rapid and broad-based detection, minimal invasiveness, clinical sample usage with low specimen volumes, high sensitivity and specificity for the immediate initiation of targeted antibiotic use in the presence of signs and symptoms of systemic inflammation, and detection of drug resistance and unknown and emerging pathogens. So far, blood culture is commonly known as the gold standard for the detection of microbial pathogens in the bloodstream. However, the organisms’ growth to detectable levels in routine blood cultures can take up to 5 days, with additional time being required for identification (24 h) and testing for antibiotic susceptibility (48 h) [111,112,113,114]. False positives via contamination during sample collection (e.g., *Staphylococcus epidermidis*) are also common. Moreover, adults with bacteremia and/or with fungemia might receive an inappropriate treatment before microbiology culture results become available [115].

The delayed detection of pathogens and the prolonged and empiric use of broad-spectrum antibiotics might expose patients to adverse events such as antibiotic allergic reactions and drug toxicity, antimicrobial-resistant bacterial strain selection, longer hospitalization time, and increased medical bills [116]. Following this evidence, although routine blood culture is still considered the gold standard diagnostic for sepsis, it is essential to shorten as well as to improve current laboratory procedures for the detection of microorganisms.

Besides blood culture techniques, a new generation of technologies for sepsis diagnosis, which are not dependent upon initial microbial growth, are emerging. Amongst them, nucleic acid amplification technologies (NAATs) amplify the nucleic acid sequences to a detectable level and identify the infecting agent or the status of the immune response. The detection of bacterial DNA fragments by real-time polymerase chain reaction (RT-PCR) in blood samples and the detection of 16S rRNA fragments of Gram-positive and Gram-negative bacteria or 18S rRNA fragments of *Candida* spp. appear to be very promising for shortening pathogen identification, since they have shown a high degree of specificity and sensitivity, and thus decreasing mortality and length of hospitalization and ICU stay of patients. The main disadvantages of these techniques are high costs, lack of standardization, and the need for skilled personnel to perform them [117].

Because of the complexity of sepsis pathophysiology and limitations of diagnostic tests based on blood cultures, there is great interest in promising biomarkers that could be used to effectively diagnose sepsis and to improve the prediction of mortality, especially in the early phase of the inflammatory response. Biomarkers are defined as “characteristics that are objectively measured and evaluated as an indicator of normal biological processes, pathogenic processes, or pharmacologic responses to a therapeutic intervention” and are commonly used to differentiate between distinct pathogenic conditions, indicate disease severity guide treatments, monitor therapeutic responses, and predict prognosis [118]. C-reactive protein (CRP) is probably the most widely measured acute-phase liver-produced protein in routine biochemistry. Although meta-analyses demonstrated that CRP has a moderate degree of sensitivity [119,120], not enough data are available to strongly recommend it in clinical practice. There are many causes for elevated CRP levels other than sepsis, including inflammation, burn injuries, cardiovascular disease, and malignancy, which all contribute to the low specificity and limited usage of CRP as a sepsis biomarker [121].

CD64 is an IgG-binding receptor expressed by neutrophils, monocytes, and macrophages in response to cytokines released during bacterial infection; given that its expression is upregulated in the early stages of activation of the innate immune response, it performs particularly well as a diagnostic marker of sepsis [122], although its use might be limited by flow cytometry, the method used for CD64 detection, requiring specialized laboratory equipment.

Presepsin (PSEP) is a 13 kDa soluble fragment of CD14, a glycoprotein that is expressed on the surface of immune cells, such as monocytes and macrophages, that acts as a receptor for LPS. It is filtered out through renal glomeruli and then reabsorbed and consequently catabolized within the proximal tubular cells (an inverse correlation between PSEP and glomerular filtrate rate has been described). Indeed, PSEP concentration increases when there is PAMP recognition during infection; thus, it could be helpful in differentiating between bacterial infections and non-infectious sepsis. In the immune response to sepsis, the serum levels of PSEP do increase before the ones for procalcitonin or IL-6, so it has been proposed as a potential biomarker of infection and systemic inflammation, with proposed cut-off levels for sepsis of 400–600 pg/mL. Used alone, as well as in conjunction with other established biomarkers, PSEP has been shown to provide useful insight to distinguish between normal and pathological conditions, graduate the disease severity, guide treatment, monitor therapeutic responses, and predict prognosis [123,124,125].

Another promising biomarker for sepsis and septic shock is proadrenomedullin (pro-ADM), a 48-amino-acid peptide derived from adrenomedullin, which is produced by vascular endothelial cells and smooth muscle cells and exerts biological effects on vasodilatation, bronchodilatation, natriuresis, cardiac contractility, and glomerular filtrate rate. Pro-ADM has been proposed as an early predictor of high mortality risk and severity of organ disfunction, as well as a clinical progression biomarker capable to identify patients at higher risk for delayed or insufficient therapy. Its diagnostic and prognostic value has been investigated in several clinical studies, which confirmed pro-ADM as a good biomarker in assessing a patient’s initial state, evolution, and prognosis. Uncertainties remain about the best cut-off for the early diagnosis of sepsis and for predicting patient prognosis, and further studies are needed to highlight several dark gaps about pro-ADM release kinetic and average half-life in the bloodstream in order to reduce the risk of false-positive or -negative results [126,127].

Overall, these biomarkers are limited in their specificity and are not sufficient to differentiate sepsis from other inflammatory processes, and no single biomarker has been approved for absolute diagnosis of sepsis. The only biomarker to achieve clinical implementation for sepsis is procalcitonin (PCT). PCT is a peptide, precursor of the hormone calcitonin, that is produced by parafollicular cells of the thyroid and by the neuroendocrine cells of the lung and the intestine. In healthy individuals, procalcitonin levels are nearly undetectable, whereas they are higher in patients with invasive bacterial infections. A PCT assay for the assessment of risk for developing severe sepsis in critically ill patients upon their first day of admission to intensive care units has been approved by the FDA. To this extent, several studies have demonstrated that PCT may accurately differentiate sepsis from noninfectious diseases and thereby contribute to early diagnosis and effective management of these conditions. However, given that PCT is strictly related to an inflammatory state, it may not be completely specific for infection [128,129].

The progress made in mass spectrometry has improved the use of proteomic and metabolomic techniques as emerging diagnostic tools for sepsis [130]. This approach has already been used to identify a panel of metabolites, described as a “biopattern”, for differentiating pediatric patients with suspected sepsis [131]. However, to date, the biomarkers found in metabolomic and proteomic studies of sepsis have not met the thresholds for clinical implementation due to several intrinsic limitations: large variability in sample collection time and preparation, data acquisition, and data analysis parameters that might result in variable metabolic and proteomic signatures.

Within noncoding RNAs, miRNAs represent another class of potential sepsis biomarkers. They are endogenous, single-stranded, noncoding RNA molecules, composed of 18–24 nucleotides that modulate gene expression at the post-transcriptional level, usually targeting messenger RNAs (mRNAs) and inducing their degradation and translation repression. Abnormal expression of a large panel of miRNAs, including miRNA-122a, -146a, and -205 family members, has been reported to contribute to the development and diagnosis of sepsis (through LPS and cytokine stimulation). In this context, a promising method is the use of specific miRNAs to detect microbiological species instead of blood cultures. This can be possible by detecting changes in the expression of miRNAs produced by microorganisms. The specificity and selectivity of the method might be increased by detecting the changes in the expression of miRNAs in various bacterial infections [132,133,134].

Finally, a different approach to sepsis diagnosis has been the development of computer-based predictive algorithms that can measure, in real time, the risk for a patient to develop sepsis three hours prior to an extended sepsis episode. The prediction itself is achieved through the analysis of correlations between nine common vital sign measurements, namely age, heart rate, oxygen saturation, pH, pulse pressure, respiration rate, temperature, systolic blood pressure, and white cell count [135]. This type of approach could be attractive due to the use of routinely measured parameters without the need for any extra testing.

## 5. Therapeutic Approach

### 5.1. General Considerations

Besides supportive therapy (vasopressor administration, mechanical ventilation, renal replacement therapy), the treatment of sepsis and septic shock is based on empirical antibiotic therapy and infection source control. The 2021 Surviving Sepsis Campaign provides recommendations about the management of sepsis and septic shock [136]. As reported above, it is crucial to collect blood cultures prior to antibiotic administration and to start therapy with broad-spectrum antibiotics within 1 h after recognition of sepsis or septic shock condition. In 2014, the MEDUSA trial showed that delay in antimicrobial therapy and source control was associated with increased mortality in sepsis and septic shock patients: every hour the antibiotic therapy was delayed, mortality increased by 2% [137,138].

Nevertheless, it is important to administer appropriate antibiotic therapy rather than early administration of any antibiotic. Kumar and colleagues demonstrated that initiation of inappropriate antimicrobials was associated with a 5-fold reduction in survival from 52.0% to 10.3% in patients with septic shock [139]. The choice of the proper antibiotic is mostly guided by the following:

- Pathogen epidemiology (patient from community, chronic care institution or hospital, and local pathogen prevalence and susceptibility);
- Presumed anatomical focus or source of infection;
- Patient’s features (concomitant underlying diseases, chronic organ failures, medications, devices, immune status, colonization with specific pathogens, and allergies).

Chosen antimicrobials must cover all likely pathogens, including bacterial and potentially fungal and viral coverage. Once the pathogen has been identified and sensitivities are established, therapy can be narrowed. Approximately one-third of patients with sepsis do not have a causative pathogen identified [136]. Since continued unnecessary antimicrobial therapy is associated with major individual and societal risks, it is recommended to de-escalate antimicrobials based on clinical improvement even if cultures are negative.

Initial therapy for a sepsis of unknown origin should cover both Gram-positive and Gram-negative bacteria. A “multidrug regimen” is usually chosen to broaden pathogen coverage, until microbiological exams reveal specific causative pathogens. Selected individuals (i.e., neutropenic patients) and hospitalized patients, characterized by a dysregulated immune system, are at high risk of infection with MDR microorganisms. Several scores have been designed to assess patients’ risk for harboring a resistant pathogen, such as those of Shorr et al. [140] and Bassetti et al. [141] (Table 1).

### 5.2. Patients in ICU

Critically ill patients with severe sepsis present a significant fluid shift from the intravascular compartment to interstitial space, caused by aggressive fluid resuscitation and hyperdynamic state associated with sepsis itself [142,143,144,145,146,147,148,149,150,151,152,153,154]. Extracellular fluid changes may also be enhanced by edematous states, pleural effusion, postsurgical drains, and extracorporeal membrane oxygenation.

Time-dependent antibiotics, which reach maximal efficacy when their concentration exceeds the MIC for a longer time, are mostly hydrophilic, so they result underdosed when Vd and drug clearance are increased. Such is the case of β-lactams, whose antimicrobial activity is optimized by more frequent administration rather than higher doses. Studies in ICUs have demonstrated that extended (3–4 h) or continuous (24 h) infusions of β-lactam antibiotics have equivalent or improved outcomes compared to intermittent (0.5–1 h) infusions, without increased adverse events.

For example, extended infusions of cefepime, administered over 3 to 4 h, were shown to significantly decrease mortality (20% for intermittent infusion vs. 3% for extended infusion; *p* = 0.03) and ICU length of stay (18.5 days for intermittent infusion vs. 8 days for extended infusion; *p* = 0.04) for invasive *Pseudomonas aeruginosa* infections. Patients treated with continuous infusions of piperacillin–tazobactam have had a better clinical response when compared to inpatients treated with intermittent infusions [139]. On the contrary, lipophilic antibiotics such as fluoroquinolones and macrolides are less affected by changes in fluid status [155].

For concentration-dependent antibiotics such as aminoglycosides or daptomycin, dose optimization can be achieved by combining peak (1 h post-infusion cessation) and trough sampling with dose optimization based on the calculated AUC. For such drugs, therapeutic drug monitoring (TDM) is a useful tool to attain C_max_/MIC target and to reduce nephrotoxicity (for aminoglycosides) which is associated with elevated trough concentrations [154].

Antibiotics such as glycopeptides (e.g., vancomycin) are both concentration- and time-dependent: their exposure target achievement is described by the area under the concentration–time curve during a 24 h time period (AUC_0–24_) to the MIC ratio (AUC_0–24_/MIC). A PK/PD target of AUC_0–24_/MIC of 400 for vancomycin is generally efficacious against organisms such as methicillin-resistant *Staphylococcus aureus* (MRSA). Besides, given that vancomycin is predominantly renally eliminated, loading doses are used in critically ill patients to overcome the lag time in achieving target antibiotic concentrations and to minimize risk of treatment failure [139].

Hypoalbuminemia also increases the Vd because an enhanced proportion of unbound drug is distributed to tissues and is available for elimination. Therefore, highly protein-bound antibiotics require altered doses to reach target concentrations: time-dependent antibiotics such as ertapenem and flucloxacillin will need more frequent dosing, while AUC_0-24_/MIC-dependent drugs such as daptomycin will need higher doses [139].

### 5.3. Patients with Altered Renal Clearance

Dose adjustment is routinely recommended in patients with impaired renal function. Nevertheless, critically ill patients often present an augmented renal clearance (ARC) with a 130–160 mL/min creatinine clearance, due to aggressive fluid resuscitation, increased cardiac output, vasopressor use, and enhanced kidney blood flow. Patients with ARC are more likely to be younger (age < 50 years), male, have a modified Sequential Organ Failure Assessment Score (SOFA < 4 or less), and be admitted because of trauma [154]. This condition requires antimicrobial dose adjustments to avoid subtherapeutic concentrations, especially for β-lactams. Unfortunately, dose increase in these patients is not standard practice.

Another condition that may profoundly affect antibiotic dosing is continuous renal replacement therapy (CRRT), used for AKI management in hemodynamically unstable critically ill patients. Indeed, severe sepsis and septic shock are among the two most common reasons for CRRT initiation. CRRT can differ in modalities, hemofilters, and effluent rates, all of which may require dosing adjustments [156]. Seyler et al. revealed that the recommended doses of β-lactams for patients receiving CRRT with *Pseudomonas aeruginosa* infection were generally not adequate to attain pharmacodynamic targets in the first 48 h of therapy [157]. Roberts et al. similarly report that usual empirical dosing of antibiotics in severely ill patients with CRRT failed to reach targets [158]. For example, meropenem has a short half-life of 1 h, and its elimination is mainly renal through glomerular filtration and tubular secretion. In CRRT, the half-life is 4.5 h, and drug clearance is determined by the volume of filtrate produced and the dialysate flow rate. In renal failure, the half-life of meropenem is increased up to 10-fold. In patients with ARC, standard meropenem regimens are insufficient in intermittent administration; prolonged (1 g q4h infused over 120 min) or continuous infusion ensures an appropriate MIC coverage over the whole dosing interval [152].

### 5.4. Obese Patients

Clinical studies report conflicting results about the impact of obesity on mortality in critically ill sepsis patients. Obesity is known as a chronic inflammation state that is related to increased oxidative stress [159]. A multitude of physiologic changes affecting PK and PD can occur in obese patients and may be responsible for antibiotic treatment failure due to lower serum concentration. In critically ill obese patients, antimicrobial Vd and clearance can be very changeable (Table 2).

In particular, Vd is generally increased as a result of increased adipose tissue (which can affect the Vd of lipophilic antimicrobials) and increased lean mass and plasma volume (which can affect the Vd of hydrophilic antimicrobials). These changes add up to all changes that can occur in other critically ill patients. Furthermore, in obese patients, renal clearance is often augmented, most likely due to the increased kidney mass and renal blood flow associated with obesity. That may affect the elimination rate. Conversely, hepatic clearance could be reduced by the great incidence of hepatic steatosis or other hepatic disfunction that can lead to a decreased drug metabolism [160]. BMI is the most common index used to classify obesity, but it considers only the weight and the height of the patients. To tailor an accurate weight-based antibiotic dosing, we need other descriptors such as total body weight (TBW), which is the actual weight of the patient and is expressed in kg; ideal body weight (IBW), which relates height–weight combination to mortality for adult men and women; and adjusted body weight (ABW), which relates the other size descriptors with a dosing weight correction factor, an index that reflects the different distribution of drugs in the adipose tissue and is different for each antibiotic, going from 0.3 to 0.4. For lipophilic antibiotics, the most important index for Vd seems to be TBW, because they can be distributed into tissues, including adipose tissues. Conversely, ABW is most appropriate for hydrophilic antimicrobials, which have limited distribution to adipose tissues.

The measurement of ClCr via 24 h urine collection is suggested to determine the correct renal clearance in obese patients. That is more important in this population because formulas such as the Cockroft–Gault equation (based on IBW) do not consider body composition and show large errors in ClCr estimates [161].

In general, if renal and hepatic functions are normal, the available data support using the high end of the dosing range, especially as regards penicillins, cephalosporins, carbapenems, and fluoroquinolones. In particular, for meropenem, the use of higher doses and prolonged infusion regimens is suggested [148,161]. For vancomycin, recent studies suggest a divided-dose strategy reducing the loading dose to 20–25 mg/kg and then adjusting the other doses based on renal function, trough levels, and possible area under the curve/MIC ratios. Two-point measurement, peak and trough, would increase the accuracy of AUC estimates. In general, if available, the use of TDM is recommended [148].

### 5.5. Burn Patients

Burn patients represent a particular population of critically ill patients. They are more susceptible to acquiring infections, and sepsis is the most important cause of mortality (rates of sepsis-related death are 50–84% in adult burn patients) [162]. This increased susceptibility has been attributed to some causes such as a nonspecific immunosuppressive state induced by burns (myeloid maturation arrest causing neutropenia, compromised cytotoxic T lymphocyte response, impaired neutrophil function, and decreased macrophage production [163]), loss of skin protection, respiratory injury from smoke, and frequent use of invasive devices (tracheal intubation, intravascular and urinary catheters) [164]. The leading cause of sepsis in these patients is the infection of burn wounds, and the most common isolated organisms are Pseudomonas aeruginosa and methicillin-resistant Staphylococcus aureus [162]. Besides, burn patients frequently require prolonged hospitalization and intensive care unit, so they can incur hospital-acquired infections, including intravascular catheter-related infections and ventilator-associated pneumonia.

Burn patients present extreme pathophysiological changes and profound alteration in PK and PD parameters. Defining infection itself can be challenging in this contest because clinical symptoms that result from the systemic inflammatory response that characterizes major burns are often the same as those of sepsis. Burn injury is characterized by a biphasic systemic response. In the first 48 h, there is a great inflammatory response that induces specific hemodynamic alterations, including increased capillary leak, splanchnic and peripheral vasoconstriction, and myocardial depression. The fluids and albumin shift into the interstitial space, resulting in relative intravascular hypovolemia, systemic hypotension, and organ hypoperfusion. In this phase, the delivery of large quantities of IV fluid is recommended to prevent further organ dysfunction and restore the circulating plasma volume. The second phase of major burn injury is characterized by a hypermetabolic state, in part mediated by elevated concentrations of endogenous catecholamines, characterized by supraphysiologic thermogenesis, increased catabolism of protein, and cardiac contractility that leads to a major organ blood flow and reduction in systemic vascular resistance. Due to the extreme physiological changes associated with major burns, the PK of antimicrobials is significantly distorted, and the application of “standard” doses is likely to result in suboptimal concentrations (either sub- or supratherapeutic) and clinical failure or drug toxicity [165,166].

In this context, it is necessary to consider the intrinsic properties of the antibiotic itself, including Vd, lipid solubility, and protein binding. While the hydrophilic molecules, such as aminoglycosides and β-lactams, normally have a relatively small Vd (principally restrict to extracellular space), this can be significantly increased in major burn injury, principally because of the widespread capillary leak, interstitial edema formation, and aggressive large volume IV fluid resuscitation. Besides, it is important to consider that the unbound fraction of a drug is responsible for the pharmacological effects and any potential toxicity, since hypoalbuminemia is often present in burn patients. Albumin binds acidic antibiotics, such as ceftriaxone, teicoplanin, daptomycin, and ertapenem. Finally, it must be noted that the renal function in burn patients is extremely variable, going from AKI requiring institution of renal replacement therapy to ARC.

Given these substantial variations in PK, alternative dosing strategies are required. It is necessary to give an adequate loading dose to rapidly achieve therapeutic concentration for an efficient bacterial killing, especially for concentration-dependent antibiotics. Regarding hydrophilic agents, the increased Vd, particularly in the case of ARC, can lead to a reduction in drug exposure with the use of “standard” doses. Good strategies that increase the probability of achieving adequate concentration are to administer the antibiotics in more frequent administration or the use of continuous infusions. For these reasons, TDM is highly recommended to optimize drug doses and dosing intervals [152].

### 5.6. Adjunctive Therapies

Since sepsis and septic shock are characterized by a dysfunction of the immune response, with an initial increase in proinflammatory cytokines and a subsequent immune-paralysis, adjunctive immune-modulatory treatments have been developed in support of antibiotic therapies to restore immune response. Single adjunctive therapies have been studied and are currently being evaluated in clinical trials, with discordant results. Nevertheless, an observational study by Marik et al. showed a synergistic effect of a combination of intravenous vitamin C, thiamine, and hydrocortisone, resulting in a reduction in organ dysfunction and mortality of patients with septic shock [167].

We report a summary of the rationale and limitations for use of each adjuvant therapy in Table 3.

## 6. Take-Home Messages

**Figure 2:**
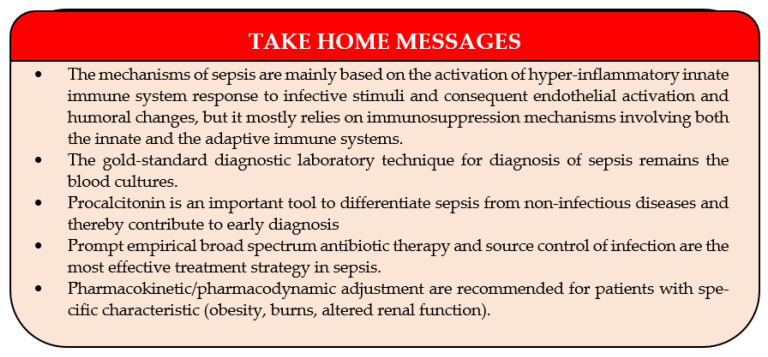
“Take home messages” about the main points treated in this review.

In Figure 2 are reported most important take home messages from this review.

- The mechanisms of sepsis are mainly based on the activation of a hyperinflammatory innate immune system response to infective stimuli and consequent endothelial activation and humoral changes, but it mostly relies on immunosuppression mechanisms involving both the innate and the adaptive immune systems.
- The gold-standard diagnostic laboratory technique for the diagnosis of sepsis remains blood cultures.
- Procalcitonin is an important tool to differentiate sepsis from noninfectious diseases and thereby contribute to early diagnosis.
- Prompt empirical broad-spectrum antibiotic therapy and source control of infection are the most effective treatment strategy in sepsis.
- Pharmacokinetic/pharmacodynamic adjustments are recommended for patients with specific characteristics (obesity, burns, altered renal function).

## 7. Conclusions

The dysregulated host response to infection leading to sepsis and septic shock is a life-threatening event that, despite advances in organ support and antimicrobial therapy, as well as the implementation of international guidelines, is still associated with a high mortality rate. This evidence warrants an urgent clarification of the molecular mechanisms underlying clinical response in patients with sepsis or septic shock.

The key to improving these processes lies in acquiring in-depth knowledge of the intricate interplay between host defense, infection, and pathogen virulence, as well as timing and type of interventions that are most effective according to the personal characteristics of individual patients. Of importance, the pharmacokinetic and pharmacodynamic properties of antibiotics should be considered because of changes in clearance and volume of distribution that are frequently observed in critically ill patients, with the potential to influence the concentration of the drug at the site of infection [186,187,188,189,190,191,192,193,194,195,196,197,198,199,200].

On these bases, the knowledge of mechanisms related to progression from sepsis to septic shock and adequate management of patients, including choice and dosages of antimicrobials, are crucial to improving the outcome of septic patients.

## Figures and Tables

**Table 1:** Main risk factors for MDR infections.

Advanced age
Diabetes
End-stage liver disease
Immunosuppressive therapy
Use of corticosteroids
Malignancy
Organ transplantation
Recent surgery
Recent exposure (<3 months) to antibiotic therapy
Prior hospital admission
MDR colonization
Local epidemiology

Legend. MDR: multidrug-resistant.

**Table 2:** Antimicrobials requiring dose adjustment in obese patients.

Antibiotic	Recommended Dosages
Piperacillin–tazobactam	Up to 4.5 g q8h (prolonged infused over 4 h) or 4.5 g q6h (30 min infusion) Prolonged infusions preferred for critically ill obese patients
Ceftazidime	Up to 2 g q8h prolonged infusion
Meropenem	Consider extended or continuous infusion Dosage adjustments based solely on body weight are unnecessary
Vancomycin	Load: 20–25 mg/kg_TBW_ Maintenance: 10–15 mg/kg_TBW_ q12h initially, then adjust by TDM Consider 10–12.5 mg/kg_TBW_ q12h if BMI ≥ 40 kg/m^2^ Alternative approach using ABW0.4: loading dose 25–30 mg/kg_ABW_, initial maintenance dose ~15 mg/kg_ABW_ q12h
Daptomycin	Same weight-based dose but use ABW_0.4_ Use of TBW to determine ClCr by the Cockcroft–Gault equation overestimated GFR in morbidly obese subjects. Use of the four-variable MDRD equation or the Cockcroft–Gault equation using IBW provided an unbiased estimate of GFR Consider TDM
Levofloxacin	750 mg q24h (over 90 min) 1000 mg q24h has been suggested for ClCr > 110 mL/min to target Gram-negative pathogens Optimization of drug exposure by TDM would be helpful
Aminoglycosides (gentamycin, amikacin)	Use ABW_0.4_ for initial dose
Clindamycin	IV: 600 mg q6h or 900 mg q8h PO: 450–600 mg q6h or 600–900 mg q8h

Legend: ABW: adjusted body weight; BMI: body mass index; ClCr: creatinine clearance; TDM: therapeutic drug monitoring.

**Table 3:** Adjunctive therapies for treatment of sepsis and septic shock.

Treatment	Rationale for Use	Limitations	References
**Corticosteroids**	The recommendation is to use iv hydrocortisone if and only in septic shock with haemodynamic instability that doesn’t respond to fluid resuscitation and vasopressor therapy. Increase adrenergic responsiveness and downregulate the exuberant anti-inflammatory response. Controversial role: most of the studies show no benefit on survival, but can result in faster haemodynamic stabilization, and fewer days on the ventilator. A study (APROCCHSS-2018) reports a significant reduction in 90-day mortality in patients treated with the association of hydrocortisone plus fludrocortisone.	Immunocompromised patients. In these patients, corticosteroids therapy had adverse effects on hemodynamic stability, prolonged ICU and hospital duration, and increased risk of hyperglycemia.	Annane 2018-APROCCHSS [168] Lu 2020 [169]
**Vitamin A** **(Retinol and** **β-carotene)**	Reduces oxidative stress and completely block the lipid peroxidation. Vitamine A (retinol and β-carotene) are depleted in patients with infections, sepsis and septic shock compared to healthy patients. β-carotene has more antioxidant properties than retinol, while retinol enhances the antioxidant effect of ascorbic acid and can quickly accumulate during supple- mentation → β-carotene (and not retinol) should be supllemented during deficiency.	Vitamin A cannot be directly measured, that is why it is needed to dose serum β-carotene and plasmatic retinol. High rate of renal excretion of retinol and retinol binding protein are detected during infection and sepsis, explaining the deficinecy but also limiting the supllementation target. Reduces mortality and ICU length of stay but not invasive support ventilation occurrence.	Zanza 2019 [170] Matos 2012 [171]
**Vitamin B1** **(Thiamine)**	Cofactor for pyruvate dehydrogenase, essential for glucose metabolism and generation of adenosine triphosphate → promotes aerobic metabolism, reduces lactate; reduces oxidative stress; reduces oxalate production from vitamin C, decreasing the risk of oxalate nephropathy. Thiamine is depleted in the hypermetabolic state of septic shock.		Hwang 2019 (ATESS) [172] Coloretti 2020 [173] Parikh 2019 [174]
**Vitamin C** **(Ascorbic acid)**	Cofactor/co-substrate for the synthesis of catecholamines (norepinephrine) and vasopressin; protective antioxidant; improves microvascular function (contributes to endothelial cell proliferation and reduces endothelial barrier permeability); has bacteriostatic activity at high concentrations. Vitamin C is depleted in septic shock and oxidative stress is elevated.	Increased metabolization into oxalic acid → calcium oxalate nephropathy; altered blood glucose measurements; increased iron absorption; false negative results on fecal occult blood testing; hemolysis in patients with G6PD enzyme deficiency; uncertainty regarding optimal dose and timing; many trials show no efficacy when administered alone, but better outcome when administer with hydrocortisone and thiamine.	Hwang 2019 (ATESS) [172] Coloretti 2020 [169] Kuhn 2019 [175] Moskowitz 2018 [176] Ahn 2019 [177]
**Vitamin E**	Antioxidant activities; stabilization of cellular membranes; empowerment of the immune response during infections. In most studies about the use of vitamin E in critical care settings it is not used as monotherapy but in combination with other antioxidant compounds.	Unconclusive results from clinical trial.	Zanza 2019 [170]
**Intravenous immunoglobulins (IVIg)**	Pathogens and toxins clearance; anti-inflammatory effects at high doses; antiapoptotic effect; endogenous immunoglobulins are decreased in sepsis and septic shock.	Rare allergic reactions; renal failure; cholestasis; different outcomes in different infections and pathogens; uncertainty about effects of different IVIg composition (percentage of IgG, IgM or IgA); no studies have compared IVIg regimens to one another; expensive.	Busani 2017 [178] Aubron 2019 [179] Iizuka 2017 [180]
**Extracorporeal Blood Purification and Hemoadsorption (CytoSorb)**	Remove endotoxins, proinflammatory mediators and lactate; hemodynamics improvement and reduction in vasopressor doses.	Removal of drugs and useful molecules (e.g., immune mediators); should be started within 24 h after onset of septic shock.	Busani 2017 [178] Kogelmann 2017 [181] Dimski 2020 [182]
**Anti-PD-1 and** **anti-PD-L1 antibodies**	Inhibition of the PD-1/PD-1L pathway, which inhibits T cells activation by blocking CD28 signalling. Antiapoptotic effect; increase in T cell IFNγ production.	Rare autoimmune reactions; lack of in vivo studies in septic shock patients.	Busani 2017 [178] Thampy 2018 [183]
**OX-40L**	Ligand of OX-40L (on T lymphocytes surface) which activates T cells through the NK-kB and PI3 kinase pathways. Antiapoptotic effect; increase in T cell IFNγ production.	lack of in vivo studies in septic shock patients.	Thampy 2018 [183]
**IL-7 and IL-15**	Antiapoptotic effect; expansion and activation of T and NK cells; production of proinflammatory cytokines.	Fever; hypotension; capillary leak syndrome; evidence only from animal models of septic shock.	Busani 2017 [178] Thampy 2018 [183]
**Adrecizumab**	Adrenomedullin (ADM) non-neutralizing antibody; ADM is a vasodilator peptide which can contribute to hypotension in sepsis, but it also stabilises endothelial barrier; excessive levels of circulating ADM in sepsis are related to higher mortality; binding circulating ADM, adrecizumab potentiates ADM function of keeping vascular integrity and reduces ADM vasodilator activity.	Benefit demonstrated on animal models of septic shock; trials on humans still ongoing; may need to administer within 12 h following vasopressor therapy; available data limited to patients with high levels of ADM.	Geven 2019 [184] Deniau 2020 [185]
**Selective digestive tract decontamination**	Use of topical and systemic antimicrobial to reduce the gut abundance of potentially pathigenic microorganisms. Reduction of respiratory tract infections and mortality among adults receiving mechanical ventilation. Eradication and less acquisition of rectal resistant microorganisms.	Limited use related to concern on promotion of antibiotic resistant strains development.	Kullberg 2021 [89]
**Probiotics**	Alive bacteria of selected species and strains. Alteration of gut microbiome to reverse the disruption in alha diversity and reduce the abundances of pathogenic bacteria.	Limited evindence of efficacy, probably to relate with the high variability between probiotic formulations and between bacterial strains phenotype of the same specie.	Kullberg 2021 [89]
**Fecal microbiome transplant**	Intrarectal transplant of feces from a healthy donor to a ill patient. It provide a complete microbiome and has been show to cure Clostridioided difficile colitis and few cases of sepsis. No randomized clinical trial results available.	Risk of transference of antibiotic resistant strain.	Kullberg 2021 [89]

## References

[1] Reinhart K., Daniels R., Kissoon N., Machado F.R., Schachter R.D., Finfer S. (2017). Recognizing Sepsis as a Global Health Priority—A WHO Resolution. N. Engl. J. Med..

[2] World Health Organization (2017). Improving the Prevention, Diagnosis and Clinical Management of Sepsis. http://apps.who.int/gb/ebwha/pdf_files/WHA70/A70_R7-en.pdf.

[3] Weinstein M.P., Reller L.B., Murphy J.R., Lichtenstein K.A. (1983). The Clinical Significance of Positive Blood Cultures: A Comprehensive Analysis of 500 Episodes of Bacteremia and Fungemia in Adults. I. Laboratory and Epidemiologic Observations. Clin. Infect. Dis..

[4] Lee C.-C., Chen S.-Y., Chang I.-J., Chen S.-C., Wu S.-C. (2007). Comparison of Clinical Manifestations and Outcome of Community-Acquired Bloodstream Infections Among the Oldest Old, Elderly, and Adult Patients. Medicine.

[5] Weinstein M.P., Towns M.L., Quartey S.M., Mirrett S., Reimer L.G., Parmigiani G., Reller L.B. (1997). The Clinical Significance of Positive Blood Cultures in the 1990s: A Prospective Comprehensive Evaluation of the Microbiology, Epidemiology, and Outcome of Bacteremia and Fungemia in Adults. Clin. Infect. Dis..

[6] Elixhauser A., Friedman B., Stranges E. (2011). Septicemia in U.S. Hospitals, 2009: Statistical Brief #122. Healthcare Cost and Utilization Project (HCUP) Statistical Briefs.

[7] Buehler S.S., Madison B., Snyder S.R., Derzon J., Cornish N.E., Saubolle M.A., Weissfeld A.S., Weinstein M.P., Liebow E.B., Wolk D.M. (2016). Effectiveness of Practices to Increase Timeliness of Providing Targeted Therapy for Inpatients with Bloodstream Infections: A Laboratory Medicine Best Practices Systematic Review and Meta-analysis. Clin. Microbiol. Rev..

[8] Geroulanos S., Douka E.T. (2006). Historical perspective of the word “sepsis”. Intensiv. Care Med..

[9] Singer M., Deutschman C.S., Seymour C.W., Shankar-Hari M., Annane D., Bauer M., Bellomo R., Bernard G.R., Chiche J.-D., Coopersmith C.M. (2016). The Third International Consensus Definitions for Sepsis and Septic Shock (Sepsis-3). JAMA.

[10] Evans T. (2018). Diagnosis and management of sepsis. Clin. Med..

[11] Takeuchi O., Akira S. (2010). Pattern Recognition Receptors and Inflammation. Cell.

[12] Schroder K., Tschopp J. (2010). The Inflammasomes. Cell.

[13] Remick D.G. (2007). Pathophysiology of Sepsis. Am. J. Pathol..

[14] Carroll M.C., Isenman D.E. (2012). Regulation of Humoral Immunity by Complement. Immunity.

[15] Sacks S.H., Zhou W. (2012). The role of complement in the early immune response to transplantation. Nat. Rev. Immunol..

[16] Lambris J.D., Ricklin D., Geisbrecht B.V. (2008). Complement evasion by human pathogens. Nat. Rev. Genet..

[17] Markiewski M.M., Lambris J.D. (2007). The Role of Complement in Inflammatory Diseases from Behind the Scenes into the Spotlight. Am. J. Pathol..

[18] Guo R.-F., Ward P.A. (2005). Role of C5A in inflammatory responses. Annu. Rev. Immunol..

[19] Wessels M.R., Butko P., Ma M., Warren H.B., Lage A.L., Carroll M.C. (1995). Studies of group B streptococcal infection in mice deficient in complement component C3 or C4 demonstrate an essential role for complement in both innate and acquired immunity. Proc. Natl. Acad. Sci. USA.

[20] Flierl M.A., Rittirsch D., Nadeau B.A., Day D.E., Zetoune F.S., Sarma J.V., Huber-Lang M.S., Ward P.A. (2008). Functions of the complement components C3 and C5 during sepsis. FASEB J..

[21] Ward P.A. (2008). Role of the complement in experimental sepsis. J. Leukoc. Biol..

[22] Huber-Lang M.S., Younkin E.M., Sarma J.V., McGuire S.R., Lu K.T., Guo R.F., Padgaonkar V.A., Curnutte J.T., Erickson R., Ward P.A. (2002). Complement-Induced Impairment of Innate Immunity During Sepsis. J. Immunol..

[23] Tomhave E.D., Richardson R.M., Didsbury J.R., Menard L., Snyderman R., Ali H. (1994). Cross-desensitization of receptors for peptide chemoattractants. Characterization of a new form of leukocyte regulation. J. Immunol..

[24] Riedemann N.C., Guo R.-F., Bernacki K.D., Reuben J.S., Laudes I.J., Neff T.A., Gao H., Speyer C., Sarma V.J., Zetoune F.S. (2003). Regulation by C5a of Neutrophil Activation during Sepsis. Immunity.

[25] Riedemann N.C., Guo R., Laudes I.J., Keller K., Sarma V.J., Padgaonkar V., Zetoune F.S., Ward P.A. (2002). C5a receptor and thymocyte apoptosis in sepsis. FASEB J..

[26] Hangen D.H., Stevens J.H., Satoh P.S., Hall E.W., O’Hanley P.T., Raffin T.A. (1989). Complement levels in septic primates treated with anti-C5a antibodies. J. Surg. Res..

[27] Müller-Redetzky H., Kellermann U., Wienhold S.-M., Gutbier B., Lienau J., Hellwig K., Reppe K., Letsiou E., Tschernig T., Scholz M. (2020). Neutralizing Complement C5a Protects Mice with Pneumococcal Pulmonary Sepsis. Anesthesiol..

[28] Deutschman C.S., Tracey K.J. (2014). Sepsis: Current Dogma and New Perspectives. Immunity.

[29] Joffre J., Hellman J., Ince C., Ait-Oufella H. (2020). Endothelial Responses in Sepsis. Am. J. Respir. Crit. Care Med..

[30] Schouten M., Wiersinga W.J., Levi M., Van Der Poll T. (2007). Inflammation, endothelium, and coagulation in sepsis. J. Leukoc. Biol..

[31] Opal S.M., Van Der Poll T. (2015). Endothelial barrier dysfunction in septic shock. J. Intern. Med..

[32] Weinbaum S., Tarbell J.M., Damiano E.R. (2007). The Structure and Function of the Endothelial Glycocalyx Layer. Annu. Rev. Biomed. Eng..

[33] Chelazzi C., Villa G., Mancinelli P., De Gaudio A.R., Adembri C. (2015). Glycocalyx and sepsis-induced alterations in vascular permeability. Crit. Care.

[34] Rubio-Gayosso I., Platts S.H., Duling B.R. (2006). Reactive oxygen species mediate modification of glycocalyx during ischemia-reperfusion injury. Am. J. Physiol. Circ. Physiol..

[35] Bone R.C. (1991). The Pathogenesis of Sepsis. Ann. Intern. Med..

[36] Karpman D., Ståhl A.-L., Arvidsson I., Johansson K., Loos S., Tati R., Békássy Z., Kristoffersson A.-C., Mossberg M., Kahn R. (2015). Complement Interactions with Blood Cells, Endothelial Cells and Microvesicles in Thrombotic and Inflammatory Conditions. Immune Responses Biosurfaces.

[37] Matsuzawa-Ishimoto Y., Hwang S., Cadwell K. (2018). Autophagy and Inflammation. Annu. Rev. Immunol..

[38] Choi Y., Bowman J.W., Jung J.U. (2018). Autophagy during viral infection—A double-edged sword. Nat. Rev. Microbiol..

[39] Oami T., Watanabe E., Hatano M., Teratake Y., Fujimura L., Sakamoto A., Ito C., Toshimori K., Swanson P.E., Oda S. (2018). Blocking Liver Autophagy Accelerates Apoptosis and Mitochondrial Injury in Hepatocytes and Reduces Time to Mortality in a Murine Sepsis Model. Shock.

[40] Karagiannidis I., Kataki A., Glustianou G., Memos N., Papalois A., Alexakis N., Zografos G.C., Konstadoulakis M.M. (2016). Extended Cytoprotective Effect of Autophagy in the Late Stages of Sepsis and Fluctuations in Signal Transduction Pathways in a Rat Experimental Model of Kidney Injury. Shock..

[41] Kim M.J., Kim E.H., Pun N.T., Chang J.-H., Kim J.-A., Jeong J.-H., Choi D.Y., Kim S.-H., Park P.-H. (2017). Globular Adiponectin Inhibits Lipopolysaccharide-Primed Inflammasomes Activation in Macrophages via Autophagy Induction: The Critical Role of AMPK Signaling. Int. J. Mol. Sci..

[42] Wu H.-M., Wang J., Zhang B., Fang L., Xu K., Liu R.-Y. (2016). CpG-ODN promotes phagocytosis and autophagy through JNK/P38 signal pathway in Staphylococcus aureus-stimulated macrophage. Life Sci..

[43] Piquereau J., Godin R., Deschênes S., Bessi V.L., Mofarrahi M., Na Hussain S., Burelle Y. (2013). Protective role of PARK2/Parkin in sepsis-induced cardiac contractile and mitochondrial dysfunction. Autophagy.

[44] Yen Y.-T., Yang H.-R., Lo H.-C., Hsieh Y.-C., Tsai S.-C., Hong C.-W., Hsieh C.-H. (2013). Enhancing autophagy with activated protein C and rapamycin protects against sepsis-induced acute lung injury. Surgery.

[45] Ho J., Yu J., Wong S.H., Zhang L., Liu X., Wong W.T., Leung C., Choi G., Wang M.H., Gin T. (2016). Autophagy in sepsis: Degradation into exhaustion?. Autophagy.

[46] Takahashi W., Watanabe E., Fujimura L., Watanabe-Takano H., Yoshidome H., Swanson P.E., Tokuhisa T., Oda S., Hatano M. (2013). Kinetics and protective role of autophagy in a mouse cecal ligation and puncture-induced sepsis. Crit. Care.

[47] Hsiao H.-W., Tsai K.-L., Wang L.-F., Chen Y.-H., Chiang P.-C., Chuang S.-M., Hsu C. (2012). The Decline of Autophagy Contributes to Proximal Tubular Dysfunction During Sepsis. Shock.

[48] Su Y., Qu Y., Zhao F., Li H., Mu D., Li X. (2015). Regulation of autophagy by the nuclear factor κB signaling pathway in the hippocampus of rats with sepsis. J. Neuroinflammat..

[49] Giegerich A.K., Kuchler L., Sha L.K., Knape T., Heide H., Wittig I., Behrends C., Brüne B., Von Knethen A. (2014). Autophagy-dependent PELI3 degradation inhibits proinflammatory IL1B expression. Autophagy.

[50] Ying L., Zhao G.-J., Wu Y., Ke H.-L., Hong G.-L., Zhang H., Dong N., Wu Y., Yao Y.-M., Lu Z.-Q. (2017). Mitofusin 2 Promotes Apoptosis of CD4+ T Cells by Inhibiting Autophagy in Sepsis. Mediat. Inflamm..

[51] Oami T., Watanabe E., Hatano M., Sunahara S., Fujimura L., Sakamoto A., Ito C., Toshimori K., Oda S. (2017). Suppression of T Cell Autophagy Results in Decreased Viability and Function of T Cells Through Accelerated Apoptosis in a Murine Sepsis Model. Crit. Care Med..

[52] Lee J.P.W., Foote A., Fan H., de Castro C.P., Lang T., Jones S.A., Gavrilescu N., Mills K.H.G., Leech M., Morand E.F. (2016). Loss of autophagy enhances MIF/macrophage migration inhibitory factor release by macrophages. Autophagy.

[53] Qiu P., Liu Y., Zhang J. (2019). Review: The Role and Mechanisms of Macrophage Autophagy in Sepsis. Inflammation.

[54] Park S.Y., Shrestha S., Youn Y.-J., Kim J.-K., Kim S.-Y., Kim H.J., Park S.-H., Ahn W.-G., Kim S., Lee M.G. (2017). Autophagy Primes Neutrophils for Neutrophil Extracellular Trap Formation during Sepsis. Am. J. Respir. Crit. Care Med..

[55] Thiessen S.E., Derese I., Derde S., Dufour T., Pauwels L., Bekhuis Y., Pintelon I., Martinet W., Berghe G.V.D., Vanhorebeek I. (2017). The Role of Autophagy in Critical Illness-induced Liver Damage. Sci. Rep..

[56] Pan P., Wang X., Liu D. (2018). The potential mechanism of mitochondrial dysfunction in septic cardiomyopathy. J. Int. Med Res..

[57] Kim M.-J., Bae S.H., Ryu J., Kwon Y., Oh J.-H., Kwon J., Moon J.-S., Kim K., Miyawaki A., Lee M.G. (2016). SESN2/sestrin2 suppresses sepsis by inducing mitophagy and inhibiting NLRP3 activation in macrophages. Autophagy.

[58] Zhou Z., You Z. (2016). Mesenchymal Stem Cells Alleviate LPS-Induced Acute Lung Injury in Mice by MiR-142a-5p-Controlled Pulmonary Endothelial Cell Autophagy. Cell. Physiol. Biochem..

[59] Si X., Cao D., Chen J., Nie Y., Jiang Z., Chen M., Wu J., Guan X. (2018). miR-23a downregulation modulates the inflammatory response by targeting ATG12-mediated autophagy. Mol. Med. Rep..

[60] Jiang L., Wang M., Sun R., Lin Z., Liu R., Cai H., Tang Z., Zhang R. (2021). Methylation of miR-19b-3p promoter exacerbates inflammatory responses in sepsis-induced ALI via targeting KLF. Cell Biol. Int..

[61] Su J., Ding L. (2021). Upregulation of miR-126 inhibits podocyte injury in sepsis via EGFL6/DKC1 signaling pathway. Mol. Med. Rep..

[62] Munoz C., Carlet J., Fitting C., Misset B., Blériot J.P., Cavaillon J.M. (1991). Dysregulation of in vitro cytokine production by monocytes during sepsis. J. Clin. Investig..

[63] Delano M., Scumpia P.O., Weinstein J.S., Coco D., Nagaraj S., Kelly-Scumpia K.M., O’Malley K.A., Wynn J., Antonenko S., Al-Quran S.Z. (2007). MyD88-dependent expansion of an immature GR-1+CD11b+ population induces T cell suppression and Th2 polarization in sepsis. J. Exp. Med..

[64] Hotchkiss R.S., Coopersmith C.M., McDunn J., Ferguson T.A. (2009). The sepsis seesaw: Tilting toward immunosuppression. Nat. Med..

[65] Hotchkiss R.S., Monneret G., Payen D. (2013). Sepsis-induced immunosuppression: From cellular dysfunctions to immunotherapy. Nat. Rev. Immunol..

[66] Munfod R.S., Pugin J. (2001). Normal Responses to Injury Prevent Systemic Inflammation and Can Be Immunosuppressive. Am. J. Respir. Crit. Care Med..

[67] Xiao W., Mindrinos M.N., Seok J., Cuschieri J., Cuenca A.G., Gao H., Hayden D.L., Hennessy L., Moore E.E., Minei J.P. (2011). A genomic storm in critically injured humans. J. Exp. Med..

[68] Stearns-Kurosawa D.J., Osuchowski M.F., Valentine C., Kurosawa S., Remick D.G. (2011). The Pathogenesis of Sepsis. Annu. Rev. Pathol. Mech. Dis..

[69] Drifte G., Dunn-Siegrist I., Tissières P., Pugin J. (2013). Innate Immune Functions of Immature Neutrophils in Patients with Sepsis and Severe Systemic Inflammatory Response Syndrome. Crit. Care Med..

[70] Tamayo E., Gómez E., Bustamante J., Gómez-Herreras J.I., Fonteriz R., Bobillo F., Bermejo-Martin J.F., Castrodeza J., Heredia M., Fierro I. (2012). Evolution of neutrophil apoptosis in septic shock survivors and nonsurvivors. J. Crit. Care.

[71] Alves-Filho J.C., Spiller F., Cunha F.Q. (2010). Neutrophil paralysis in sepsis. Shock.

[72] Kasten K., Muenzer J.T., Caldwell C.C. (2010). Neutrophils are significant producers of IL-10 during sepsis. Biochem. Biophys. Res. Commun..

[73] Pillay J., Kamp V.M., Van Hoffen E., Visser T., Tak T., Lammers J.-W., Ulfman L.H., Leenen L., Pickkers P., Koenderman L. (2012). A subset of neutrophils in human systemic inflammation inhibits T cell responses through Mac-1. J. Clin. Investig..

[74] Ortiz J.A., Maroun-Eid C., Martin-Quiros A., Toledano V., Cubillos-Zapata C., Gómez-Campelo P., Varela-Serrano A., Casas-Martin J., Llanos-González E., Alvarez E. (2017). PD-L1 Overexpression During Endotoxin Tolerance Impairs the Adaptive Immune Response in Septic Patients via HIF1α. J. Infect. Dis..

[75] Ishii M., Wen H., Corsa C., Liu T., Coelho A.L., Allen R.M., Carson W.F., Cavassani K.A., Li X., Lukacs N.W. (2009). Epigenetic regulation of the alternatively activated macrophage phenotype. Blood.

[76] Rossato M., Curtale G., Tamassia N., Castellucci M., Mori L., Gasperini S., Mariotti B., De Luca M., Mirolo M., Cassatella M.A. (2012). IL-10-induced microRNA-187 negatively regulates TNF-, IL-6, and IL-12p40 production in TLR4-stimulated monocytes. Proc. Natl. Acad. Sci. USA.

[77] Cavaillon J.-M., Adib-Conquy M. (2006). Bench-to-bedside review: Endotoxin tolerance as a model of leukocyte reprogramming in sepsis. Crit. Care.

[78] Biswas S.K., Lopez-Collazo E. (2009). Endotoxin tolerance: New mechanisms, molecules and clinical significance. Trends Immunol..

[79] Monneret G., Finck M.-E., Venet F., Debard A.-L., Bohé J., Bienvenu J., Lepape A. (2004). The anti-inflammatory response dominates after septic shock: Association of low monocyte HLA-DR expression and high interleukin-10 concentration. Immunol. Lett..

[80] Hynninen M., Pettilä V., Takkunen O., Orko R., Jansson S.-E., Kuusela P., Renkonen R., Valtonen M. (2003). Predictive Value of Monocyte Histocompatibility Leukocyte Antigen-DR Expression and Plasma Interleukin-4 and -10 Levels in Critically Ill Patients with Sepsis. Shock.

[81] Guisset O., Dilhuydy M.-S., Thiébaut R., Lefèvre J., Camou F., Sarrat A., Gabinski C., Moreau J.-F., Blanco P. (2007). Decrease in circulating dendritic cells predicts fatal outcome in septic shock. Intensiv. Care Med..

[82] Dreschler K., Bratke K., Petermann S., Thamm P., Kuepper M., Virchow J.C., Lommatzsch M. (2012). Altered Phenotype of Blood Dendritic Cells in Patients with Acute Pneumonia. Respiration.

[83] Monneret G., Lepape A., Voirin N., Bohé J., Venet F., Debard A.-L., Thizy H., Bienvenu J., Gueyffier F., Vanhems P. (2006). Persisting low monocyte human leukocyte antigen-DR expression predicts mortality in septic shock. Intensiv. Care Med..

[84] Hotchkiss R.S., Tinsley K.W., Swanson P.E., Schmieg R.E., Hui J.J., Chang K.C., Osborne D.F., Freeman B.D., Cobb J.P., Buchman T. (2001). Sepsis-Induced Apoptosis Causes Progressive Profound Depletion of B and CD4+T Lymphocytes in Humans. J. Immunol..

[85] Liu Q., Li C.-S. (2017). Programmed Cell Death-1/Programmed Death-ligand 1 Pathway: A New Target for Sepsis. Chin. Med. J..

[86] Guignant C., Lepape A., Huang X., Kherouf H., Denis L., Poitevin F., Malcus C., Chéron A., Allaouchiche B., Gueyffier F. (2011). Programmed death-1 levels correlate with increased mortality, nosocomial infection and immune dysfunctions in septic shock patients. Crit. Care.

[87] Venet F., Davin F., Guignant C., Larue A., Cazalis M.-A., Darbon R., Allombert C., Mougin B., Malcus C., Poitevin-Later F. (2010). Early Assessment of Leukocyte Alterations at Diagnosis of Septic Shock. Shock.

[88] Pachot A., Monneret G., Voirin N., Leissner P., Venet F., Bohé J., Payen D., Bienvenu J., Mougin B., Lepape A. (2005). Longitudinal study of cytokine and immune transcription factor mRNA expression in septic shock. Clin. Immunol..

[89] Kullberg R.F., Wiersinga W.J., Haak B.W. (2021). Gut microbiota and sepsis: From pathogenesis to novel treatments. Curr. Opin. Gastroenterol..

[90] Adelman M.W., Woodworth M.H., Langelier C., Busch L.M., Kempker J.A., Kraft C.S., Martin G.S. (2020). The gut microbiome’s role in the development, maintenance, and outcomes of sepsis. Crit. Care.

[91] Haak B.W., Argelaguet R., Kinsella C.M., Kullberg R.F.J., Lankelma J.M., Deijs M., Klein M., Jebbink M.F., Hugenholtz F., Kostidis S. (2021). Integrative Transkingdom Analysis of the Gut Microbiome in Antibiotic Perturbation and Critical Illness. mSystems.

[92] Tourelle K.M., Boutin S., Weigand M.A., Schmitt F.C.F. (2021). Sepsis and the Human Microbiome. Just Another Kind of Organ Failure? A Review. J. Clin. Med..

[93] Ma Z., Ni G., Damania B. (2018). Innate Sensing of DNA Virus Genomes. Annu. Rev. Virol..

[94] McKernan D.P. (2020). Pattern recognition receptors as potential drug targets in inflammatory disorders. Adv. Protein Chem. Struct. Biol..

[95] Chen I.-Y., Ichinohe T. (2015). Response of host inflammasomes to viral infection. Trends Microbiol..

[96] Gu S., Chen Y., Wu Z., Chen Y., Gao H., Lv L., Guo F., Zhang X., Luo R., Huang C. (2020). Alterations of the Gut Microbiota in Patients with Coronavirus Disease 2019 or H1N1 Influenza. Clin. Infect. Dis..

[97] Zuo T., Zhang F., Lui G.C.Y., Yeoh Y.K., Li A.Y.L., Zhan H., Wan Y., Chung A.C.K., Cheung C.P., Chen N. (2020). Alterations in Gut Microbiota of Patients With COVID-19 During Time of Hospitalization. Gastroenterology.

[98] Viana S.D., Nunes S., Reis F. (2020). ACE2 imbalance as a key player for the poor outcomes in COVID-19 patients with age-related comorbidities—Role of gut microbiota dysbiosis. Ageing Res. Rev..

[99] Stoma I., Littmann E.R., Peled J.U., Giralt S., Brink M.R.M.V.D., Pamer E.G., Taur Y. (2020). Compositional Flux Within the Intestinal Microbiota and Risk for Bloodstream Infection with Gram-negative Bacteria. Clin. Infect. Dis..

[100] Freedberg D.E., Zhou M.J., Cohen M.E., Annavajhala M., Khan S., Moscoso D.I., Brooks C., Whittier S., Chong D.H., Uhlemann A.-C. (2018). Pathogen colonization of the gastrointestinal microbiome at intensive care unit admission and risk for subsequent death or infection. Intensiv. Care Med..

[101] Galley H.F. (2011). Oxidative stress and mitochondrial dysfunction in sepsis. Br. J. Anaesth..

[102] Cornaglia G., Giamarellou H., Rossolini G.M. (2011). Metallo-β-lactamases: A last frontier for β-lactams?. Lancet Infect. Dis..

[103] Magiorakos A.-P., Srinivasan A., Carey R.B., Carmeli Y., Falagas M.E., Giske C.G., Harbarth S., Hindler J.F., Kahlmeter G., Olsson-Liljequist B. (2012). Multidrug-resistant, extensively drug-resistant and pandrug-resistant bacteria: An international expert proposal for interim standard definitions for acquired resistance. Clin. Microbiol. Infect..

[104] Jones R.N. (2001). Resistance Patterns Among Nosocomial Pathogens. Chest.

[105] Akella K., Joshi G., Ibrahim S., Rutka P., Chow P., Fernando R., Sklarek H. (2019). #1274: Degree of multidrug resistance in sepsis is associated with increased in-hospital morbidity. Crit. Care Med..

[106] Knaus W.A., Draper E.A., Wagner D.P., Zimmerman J.E. (1985). APACHE II: A severity of disease classification system. Crit. Care Med..

[107] Karamouzos V., Giamarellos-Bourboulis E.J., Velissaris D., Gkavogianni T., Gogos C. (2021). Cytokine production and outcome in MDR versus non-MDR gram-negative bacteraemia and sepsis. Infect. Dis..

[108] Lee S.J., You J.S., Gharbi A., Kim Y.J., Lee M.S., Kim D.H., Lee K.W., Jung I.D., Park Y.M. (2021). IOX1 activity as sepsis therapy and an antibiotic against multidrug-resistant bacteria. Sci. Rep..

[109] Liu V.X., Fielding-Singh V., Greene J.D., Baker J.M., Iwashyna T.J., Bhattacharya J., Escobar G.J. (2017). The Timing of Early Antibiotics and Hospital Mortality in Sepsis. Am. J. Respir. Crit. Care Med..

[110] Husabø G., Nilsen R.M., Flaatten H., Solligård E., Frich J.C., Bondevik G.T., Braut G.S., Walshe K., Harthug S., Hovlid E. (2020). Early diagnosis of sepsis in emergency departments, time to treatment, and association with mortality: An observational study. PLoS ONE.

[111] Riedel S., Carroll K.C. (2016). Early Identification and Treatment of Pathogens in Sepsis: Molecular Diagnostics and Antibiotic Choice. Clin. Chest Med..

[112] Bacconi A., Richmond G.S., Baroldi M.A., Laffler T.G., Blyn L.B., Carolan H.E., Frinder M.R., Toleno D.M., Metzgar D., Gutierrez J.R. (2014). Improved Sensitivity for Molecular Detection of Bacterial and Candida Infections in Blood. J. Clin. Microbiol..

[113] Opota O., Jaton K., Greub G. (2015). Microbial diagnosis of bloodstream infection: Towards molecular diagnosis directly from blood. Clin. Microbiol. Infect..

[114] Connell T.G., Rele M., Cowley D., Buttery J.P., Curtis N. (2007). How Reliable Is a Negative Blood Culture Result? Volume of Blood Submitted for Culture in Routine Practice in a Children’s Hospital. Pediatrics.

[115] Sinha M., Jupe J., Mack H., Coleman T.P., Lawrence S.M., Fraley S.I. (2018). Emerging Technologies for Molecular Diagnosis of Sepsis. Clin. Microbiol. Rev..

[116] Pliakos E.E., Andreatos N., Shehadeh F., Ziakas P.D., Mylonakis E. (2018). The Cost-Effectiveness of Rapid Diagnostic Testing for the Diagnosis of Bloodstream Infections with or without Antimicrobial Stewardship. Clin. Microbiol. Rev..

[117] Liesenfeld O., Lehman L., Hunfeld K.-P., Kost G. (2014). Molecular diagnosis of sepsis: New aspects and recent developments. Eur. J. Microbiol. Immunol..

[118] Hung S.-K., Lan H.-M., Han S.-T., Wu C.-C., Chen K.-F. (2020). Current Evidence and Limitation of Biomarkers for Detecting Sepsis and Systemic Infection. Biomedicines.

[119] Tan M., Lu Y., Jiang H., Zhang L. (2019). The diagnostic accuracy of procalcitonin and C-reactive protein for sepsis: A systematic review and meta-analysis. J. Cell. Biochem..

[120] Liu Y., Hou J.-H., Li Q., Chen K.-J., Wang S.-N., Wang J.-M. (2016). Biomarkers for diagnosis of sepsis in patients with systemic inflammatory response syndrome: A systematic review and meta-analysis. SpringerPlus.

[121] Ryoo S.M., Han K.S., Ahn S., Shin T.G., Hwang S.Y., Chung S.P., Hwang Y.J., Park Y.S., Jo Y.H., Chang H.L. (2019). The usefulness of C-reactive protein and procalcitonin to predict prognosis in septic shock patients: A multicenter prospective registry-based observational study. Sci. Rep..

[122] Cong S., Ma T., Di X., Tian C., Zhao M., Wang K. (2021). Diagnostic value of neutrophil CD64, procalcitonin, and interleukin-6 in sepsis: A meta-analysis. BMC Infect. Dis..

[123] Velissaris D., Zareifopoulos N., Karamouzos V., Karanikolas E., Pierrakos C., Koniari I., Karanikolas M. (2021). Presepsin as a Diagnostic and Prognostic Biomarker in Sepsis. Cureus.

[124] Piccioni A., Santoro M., de Cunzo T., Tullo G., Cicchinelli S., Saviano A., Valletta F., Pascale M., Candelli M., Covino M. (2021). Presepsin as Early Marker of Sepsis in Emergency Department: A Narrative Review. Medicine.

[125] Yang H.S., Hur M., Yi A., Kim H., Lee S., Kim S.-N. (2018). Prognostic value of presepsin in adult patients with sepsis: Systematic review and meta-analysis. PLoS ONE.

[126] Piccioni A., Saviano A., Cicchinelli S., Valletta F., Santoro M.C., de Cunzo T., Zanza C., Longhitano Y., Tullo G., Tilli P. (2021). Proadrenomedullin in Sepsis and Septic Shock: A Role in the Emergency Department. Medicine.

[127] Li P., Wang C., Pang S. (2021). The diagnostic accuracy of mid-regional pro-adrenomedullin for sepsis: A systematic review and meta-analysis. Minerva Anestesiol..

[128] Vijayan A.L., Ravindran S., Saikant R., Lakshmi S., Kartik R. (2017). Procalcitonin: A promising diagnostic marker for sepsis and antibiotic therapy. J. Intensiv. Care.

[129] van Oers J.A., de Jong E., Kemperman H., Girbes A.R., de Lange D.W. (2019). Diagnostic Accuracy of Procalcitonin and C-reactive Protein Is Insufficient to Predict Proven Infection: A Retrospective Cohort Study in Critically Ill Patients Fulfilling the Sepsis-3 Criteria. J. Appl. Lab. Med..

[130] Ludwig K.R., Hummon A.B. (2017). Mass spectrometry for the discovery of biomarkers of sepsis. Mol. BioSyst..

[131] Mickiewicz B., Thompson G.C., Blackwood J., Jenne C.N., Winston B.W., Vogel H.J., Joffe A.R. (2018). Biomarker Phenotype for Early Diagnosis and Triage of Sepsis to the Pediatric Intensive Care Unit. Sci. Rep..

[132] Dumache R., Rogobete A.F., Bedreag O.H., Sarandan M., Cradigati A.C., Papurica M., Dumbuleu C.M., Nartita R., Sandesc D. (2015). Use of miRNAs as Biomarkers in Sepsis. Anal. Cell. Pathol..

[133] Cossart P., Lebreton A. (2014). A trip in the “New Microbiology” with the bacterial pathogen *Listeria monocytogenes*. FEBS Lett..

[134] Dai L.-L., Gao J.-X., Zou C.-G., Ma Y.-C., Zhang K.-Q. (2015). mir-233 Modulates the Unfolded Protein Response in C. elegans during *Pseudomonas aeruginosa* Infection. PLoS Pathog..

[135] Calvert J.S., Price D.A., Chettipally U.K., Barton C.W., Feldman M.D., Hoffman J.L., Jay M., Das R. (2016). A computational approach to early sepsis detection. Comput. Biol. Med..

[136] Evans L., Rhodes A., Alhazzani W., Antonelli M., Coopersmith C.M., French C., Machado F.R., Mcintyre L., Ostermann M., Prescott H.C. (2021). Surviving Sepsis Campaign: International Guidelines for Management of Sepsis and Septic Shock. Crit. Care Med..

[137] Jarczak D., Kluge S., Nierhaus A. (2021). Sepsis—Pathophysiology and Therapeutic Concepts. Front. Med..

[138] Richter D.C., Heininger A., Brenner T., Hochreiter M., Bernhard M., Briegel J., Dubler S., Grabein B., Hecker A., Krüger W.A. (2017). Bacterial sepsis: Diagnostics and calculated antibiotic therapy. Der Anaesthesist.

[139] Denny K.J., Cotta M.O., Parker S.L., Roberts J.A., Lipman J. (2016). The use and risks of antibiotics in critically ill patients. Expert Opin. Drug Saf..

[140] Shorr A.F., Zilberberg M.D., Micek S.T., Kollef M.H. (2008). Prediction of Infection Due to Antibiotic-Resistant Bacteria by Select Risk Factors for Health Care–Associated Pneumonia. Arch. Intern. Med..

[141] Bassetti M., Carnelutti A., Peghin M. (2016). Patient specific risk stratification for antimicrobial resistance and possible treatment strategies in gram-negative bacterial infections. Expert Rev. Anti-Infect. Ther..

[142] Buckman S.A., Turnbull I.R., Mazuski J.E. (2018). Empiric Antibiotics for Sepsis. Surg. Infect..

[143] Esposito S., De Simone G., Boccia G., De Caro F., Pagliano P. (2017). Sepsis and septic shock: New definitions, new diagnostic and therapeutic approaches. J. Glob. Antimicrob. Resist..

[144] Sjövall F., Perner A., Møller M.H. (2017). Empirical mono- versus combination antibiotic therapy in adult intensive care patients with severe sepsis—A systematic review with meta-analysis and trial sequential analysis. J. Infect..

[145] Russo A., Bassetti M., Bellelli V., Bianchi L., Cattaneo F.M., Mazzocchetti S., Paciacconi E., Cottini F., Schiattarella A., Tufaro G. (2021). Efficacy of a Fosfomycin-Containing Regimen for Treatment of Severe Pneumonia Caused by Multidrug-Resistant *Acinetobacter baumannii*: A Prospective, Observational Study. Infect. Dis. Ther..

[146] Dugar S., Choudhary C., Duggal A. (2020). Sepsis and septic shock: Guideline-based management. Clevel. Clin. J. Med..

[147] Coopersmith C.M., De Backer D., Deutschman C.S., Ferrer R., Lat I., Machado F.R., Martin G.S., Martin-Loeches I., Nunnally M.E., Antonelli M. (2018). Surviving sepsis campaign: Research priorities for sepsis and septic shock. Intensiv. Care Med..

[148] Allison M.G., Heil E.L., Hayes B.D. (2017). Appropriate Antibiotic Therapy. Emerg. Med. Clin. N. Am..

[149] Williams J.M., Keijzers G., Macdonald S.P., Shetty A., Fraser J.F. (2018). Review article: Sepsis in the emergency department—Part 3: Treatment. Emerg. Med. Australas..

[150] Veiga R.P., Paiva J.-A. (2018). Pharmacokinetics–pharmacodynamics issues relevant for the clinical use of beta-lactam antibiotics in critically ill patients. Crit. Care.

[151] Ahmed N., Jen S.-P., Altshuler D., Papadopoulos J., Pham V., Dubrovskaya Y. (2018). Evaluation of Meropenem Extended Versus Intermittent Infusion Dosing Protocol in Critically Ill Patients. J. Intensiv. Care Med..

[152] Burger R., Guidi M., Calpini V., Lamoth F., Decosterd L., Robatel C., Buclin T., Csajka C., Marchetti O. (2018). Effect of renal clearance and continuous renal replacement therapy on appropriateness of recommended meropenem dosing regimens in critically ill patients with susceptible life-threatening infections. J. Antimicrob. Chemother..

[153] Nielsen E.I., Cars O., Friberg L. (2011). Pharmacokinetic/Pharmacodynamic (PK/PD) Indices of Antibiotics Predicted by a Semimechanistic PKPD Model: A Step toward Model-Based Dose Optimization. Antimicrob. Agents Chemother..

[154] Heffernan A., Sime F., Taccone F.S., Roberts J.A. (2018). How to optimize antibiotic pharmacokinetic/pharmacodynamics for Gram-negative infections in critically ill patients. Curr. Opin. Infect. Dis..

[155] Campion M., Scully G. (2018). Antibiotic Use in the Intensive Care Unit: Optimization and De-Escalation. J. Intensiv. Care Med..

[156] Shaw A.R., Chaijamorn W., Mueller B.A. (2016). We Underdose Antibiotics in Patients on CRRT. Semin. Dial..

[157] Seyler L., Cotton F., Taccone F.S., De Backer D., Macours P., Vincent J.-L., Jacobs F. (2011). Recommended β-lactam regimens are inadequate in septic patients treated with continuous renal replacement therapy. Crit. Care.

[158] Roberts D.M., Roberts J., Roberts M., Liu X., Nair P., Cole L., Lipman J., Bellomo R. (2012). Variability of antibiotic concentrations in critically ill patients receiving continuous renal replacement therapy: A multicentre pharmacokinetic study. Crit. Care Med..

[159] Trivedi V., Bavishi C., Jean R. (2015). Impact of obesity on sepsis mortality: A systematic review. J. Crit. Care.

[160] Alobaid A.S., Hites M., Lipman J., Taccone F.S., Roberts J.A. (2016). Effect of obesity on the pharmacokinetics of antimicrobials in critically ill patients: A structured review. Int. J. Antimicrob. Agents.

[161] Meng L., Mui E., Holubar M.K., Deresinski S.C. (2017). Comprehensive Guidance for Antibiotic Dosing in Obese Adults. Pharmacother. J. Hum. Pharmacol. Drug Ther..

[162] Lopez O.N., Cambiaso-Daniel J., Branski L.K., Norbury W.B., Herndon D.N. (2017). Predicting and managing sepsis in burn patients: Current perspectives. Ther. Clin. Risk Manag..

[163] Avni T., Levcovich A., Ad-El D.D., Leibovici L., Paul M. (2010). Prophylactic antibiotics for burns patients: Systematic review and meta-analysis. BMJ.

[164] Hidalgo F., Mas D., Rubio M., Garcia-Hierro P. (2016). Infections in critically ill burn patients. Med. Intensiv..

[165] Hill D.M., Sinclair S.E., Hickerson W.L. (2017). Rational Selection and Use of Antimicrobials in Patients with Burn Injuries. Clin. Plast. Surg..

[166] Udy A.A., Roberts J.A., Lipman J., Blot S. (2018). The effects of major burn related pathophysiological changes on the pharmacokinetics and pharmacodynamics of drug use: An appraisal utilizing antibiotics. Adv. Drug Deliv. Rev..

[167] Marik P.E., Khangoora V., Rivera R., Hooper M.H., Catravas J. (2017). Hydrocortisone, Vitamin C, and Thiamine for the Treatment of Severe Sepsis and Septic Shock. Chest.

[168] Annane D., Renault A., Brun-Buisson C., Megarbane B., Quenot J.-P., Siami S., Cariou A., Forceville X., Schwebel C., Martin C. (2018). Hydrocortisone plus Fludrocortisone for Adults with Septic Shock. N. Engl. J. Med..

[169] Lu X., Wang X., Gao Y., Yu S., Zhao L., Zhang Z., Zhu H., Li Y. (2021). Efficacy and safety of corticosteroids for septic shock in immunocompromised patients: A cohort study from MIMIC. Am. J. Emerg. Med..

[170] Zanza C., Thangathurai J., Audo A., Muir H.A., Candelli M., Pignataro G., Thangathurai D., Cicchinelli S., Racca F., Longhitano Y. (2019). Oxidative stress in critical care and vitamins supplement therapy: “A beneficial care enhancing”. Eur. Rev. Med. Pharmacol. Sci..

[171] Matos A.C., Souza G.G., Moreira V. (2012). Efecto de la suplementacion con vitamina A sobre la evloucion clinica. Nutrición Hospitalaria.

[172] Hwang S.Y., Park J.E., Jo I.J., Kim S., Chung S.P., Kong T., Shin J., Lee H.J., You K.M., Jo Y.H. (2019). Combination therapy of vitamin C and thiamine for septic shock in a multicentre, double-blind, randomized, controlled study (ATESS): Study protocol for a randomized controlled trial. Trials.

[173] Coloretti I., Biagioni E., Venturelli S., Munari E., Tosi M., Roat E., Brugioni L., Gelmini R., Venturelli C., Girardis M. (2020). Adjunctive therapy with vitamin c and thiamine in patients treated with steroids for refractory septic shock: A propensity matched before-after, case-control study. J. Crit. Care.

[174] Parikh R., Belok S.H., Swamy L., Reardon C.C. (2019). Adjunctive Therapies in the Management of Septic Shock. Am. J. Respir. Crit. Care Med..

[175] Kuhn S.-O., Meissner K., Mayes L.M., Bartels K. (2018). Vitamin C in sepsis. Curr. Opin. Anaesthesiol..

[176] Moskowitz A., Andersen L.W., Huang D.T., Berg K.M., Grossestreuer A.V., Marik P.E., Sherwin R.L., Hou P.C., Becker L.B., Cocchi M.N. (2018). Ascorbic acid, corticosteroids, and thiamine in sepsis: A review of the biologic rationale and the present state of clinical evaluation. Crit. Care.

[177] Ahn J.H., Oh D.K., Huh J.W., Lim C.-M., Koh Y., Hong S.-B. (2019). Vitamin C alone does not improve treatment outcomes in mechanically ventilated patients with severe sepsis or septic shock: A retrospective cohort study. J. Thorac. Dis..

[178] Busani S., Roat E., Serafini G., Mantovani E., Biagioni E., Girardis M. (2017). The Role of Adjunctive Therapies in Septic Shock by Gram Negative MDR/XDR Infections. Can. J. Infect. Dis. Med Microbiol..

[179] Aubron C., Berteau F., Sparrow R.L. (2019). Intravenous immunoglobulin for adjunctive treatment of severe infections in ICUs. Curr. Opin. Crit. Care.

[180] Iizuka Y., Sanui M., Sasabuchi Y., Lefor A.K., Hayakawa M., Saito S., Uchino S., Yamakawa K., Kudo D., Takimoto K. (2017). Low-dose immunoglobulin G is not associated with mortality in patients with sepsis and septic shock. Crit. Care.

[181] Kogelmann K., Jarczak D., Scheller M., Drüner M. (2017). Hemoadsorption by CytoSorb in septic patients: A case series. Crit. Care.

[182] Dimski T., Brandenburger T., Slowinski T., Kindgen-Milles D. (2020). Feasibility and safety of combined cytokine adsorption and continuous veno-venous hemodialysis with regional citrate anticoagulation in patients with septic shock. Int. J. Artif. Organs.

[183] Thampy L.K., Remy K.E., Walton A., Hong Z., Liu K., Liu R., Yi V.N., Burnham C.-A.D., Hotchkiss R.S. (2018). Restoration of T Cell function in multi-drug resistant bacterial sepsis after interleukin-7, anti-PD-L1, and OX-40 administration. PLoS ONE.

[184] Geven C., Blet A., Kox M., Hartmann O., Scigalla P., Zimmermann J., Marx G., Laterre P.-F., Mebazaa A., Pickkers P. (2019). A double-blind, placebo-controlled, randomised, multicentre, proof-of-concept and dose-finding phase II clinical trial to investigate the safety, tolerability and efficacy of adrecizumab in patients with septic shock and elevated adrenomedullin concentration (AdrenOSS-2). BMJ Open.

[185] Deniau B., Takagi K., Asakage A., Mebazaa A. (2021). Adrecizumab: An investigational agent for the biomarker-guided treatment of sepsis. Expert Opin. Investig. Drugs.

[186] Alessandri F., Pugliese F., Angeletti S., Ciccozzi M., Russo A., Mastroianni C.M., D’ettorre G., Venditti M., Ceccarelli G. (2020). Procalcitonin in the Assessment of Ventilator Associated Pneumonia: A Systematic Review. Advances Experimental Medicine Biology.

[187] Bassetti M., Russo A., Righi E., Dolso E., Merelli M., D’aurizio F., Sartor A., Curcio F. (2018). Role of procalcitonin in bacteremic patients and its potential use in predicting infection etiology. Expert Rev. Anti-Infect. Ther..

[188] Bassetti M., Russo A., Righi E., Dolso E., Merelli M., D’aurizio F., Sartor A., Curcio F. (2020). Role of procalcitonin in predicting etiology in bacteremic patients: Report from a large single-center experience. J. Infect. Public Health.

[189] Russo A., Cattaneo F.M., Brunetti G., Picciarella A., Russo R., Hallgass M.E., Sabetta F. (2020). Clinical features and outcome of difficult-to-treat infections in a high-intensity medical care ward. Minerva Med..

[190] Russo A., Bassetti M., Ceccarelli G., Carannante N., Losito A.R., Bartoletti M., Corcione S., Granata G., Santoro A., Giacobbe D.R. (2019). Bloodstream infections caused by carbapenem-resistant *Acinetobacter baumannii*: Clinical features, therapy and outcome from a multicenter study. J. Infect..

[191] Russo A., Giuliano S., Ceccarelli G., Alessandri F., Giordano A., Brunetti G., Venditti M. (2018). Comparison of Septic Shock Due to Multidrug-Resistant *Acinetobacter baumannii* or *Klebsiella pneumoniae* Carbapenemase-Producing K. pneumoniae in Intensive Care Unit Patients. Antimicrob. Agents Chemother..

[192] Russo A., Falcone M., Gutiérrez-Gutiérrez B., Calbo E., Almirante B., Viale P., Oliver A., Ruiz-Garbajosa P., Gasch O., Gozalo M. (2018). Predictors of outcome in patients with severe sepsis or septic shock due to extended-spectrum β-lactamase-producing Enterobacteriaceae. Int. J. Antimicrob. Agents.

[193] Bassetti M., Vena A., Russo A., Croxatto A., Calandra T., Guery B. (2018). Rational approach in the management of *Pseudomonas aeruginosa* infections. Curr. Opin. Infect. Dis..

[194] Bassetti M., Vena A., Giacobbe D.R., Falcone M., Tiseo G., Giannella M., Pascale R., Meschiari M., DiGaetano M., Oliva A. (2020). Ceftolozane/Tazobactam for treatment of severe ESBL-producing enterobacterales infections: A multicenter nationwide clinical experience (CEFTABUSE II Study). Open Forum Infect. Dis..

[195] Bassetti M., Castaldo N., Cattelan A., Mussini C., Righi E., Tascini C., Menichetti F., Mastroianni C.M., Tumbarello M., Grossi P. (2019). Ceftolozane/tazobactam for the treatment of serious *Pseudomonas aeruginosa* infections: A multicentre nationwide clinical experience. Int. J. Antimicrob. Agents.

[196] Falcone M., Bassetti M., Tiseo G., Giordano C., Nencini E., Russo A., Graziano E., Tagliaferri E., Leonildi A., Barnini S. (2020). Time to appropriate antibiotic therapy is a predictor of outcome in patients with bloodstream infection caused by KPC-producing *Klebsiella pneumoniae*. Crit. Care.

[197] Falcone M., Russo A., Iacovelli A., Restuccia G., Ceccarelli G., Giordano A., Farcomeni A., Morelli A., Venditti M. (2016). Predictors of outcome in ICU patients with septic shock caused by *Klebsiella pneumoniae* carbapenemase–producing *K. pneumoniae*. Clin. Microbiol. Infect..

[198] Bassetti M., Righi E., Carnelutti A., Graziano E., Russo A. (2018). Multidrug-resistant *Klebsiella pneumoniae*: Challenges for treatment, prevention and infection control. Expert Rev. Antiinfect. Ther..

[199] Bassetti M., Righi E., Vena A., Graziano E., Russo A., Peghin M. (2018). Risk stratification and treatment of ICU-acquired pneumonia caused by multidrug- resistant/extensively drug-resistant/pandrug-resistant bacteria. Curr. Opin. Crit. Care.

[200] Bassetti M., Russo A., Carnelutti A., La Rosa A., Righi E. (2018). Antimicrobial resistance and treatment: An unmet clinical safety need. Expert Opin. Drug Saf..

